# Cutting Through the Noise: Predictors of Successful Online Message Retransmission in the First 8 Months of the COVID-19 Pandemic

**DOI:** 10.1089/hs.2020.0200

**Published:** 2021-02-18

**Authors:** Scott Leo Renshaw, Sabrina Mai, Elisabeth Dubois, Jeannette Sutton, Carter T. Butts

**Affiliations:** Scott Leo Renshaw, MA, and Sabrina Mai are PhD Students, Department of Sociology, and Carter T. Butts, PhD, is a Professor in the Departments of Sociology, Statistics, Computer Science, and Electrical Engineering and Computers; all at the University of California Irvine, Irvine, CA. Elisabeth Dubois, MBA, is a PhD Student and Jeannette Sutton, PhD, is an Associate Professor, College of Emergency Preparedness, Homeland Security, and Cyber Security, University of Albany, SUNY, Albany, NY.

**Keywords:** COVID-19, Social media, Risk communication, Public health preparedness/response, Epidemic management/response

## Abstract

In this paper, we investigate how message construction, style, content, and the textual content of embedded images impacted message retransmission over the course of the first 8 months of the coronavirus disease 2019 (COVID-19) pandemic in the United States. We analyzed a census of public communications (n = 372,466) from 704 public health agencies, state and local emergency management agencies, and elected officials posted on Twitter between January 1 and August 31, 2020, measuring message retransmission via the number of retweets (ie, a message passed on by others), an important indicator of engagement and reach. To assess content, we extended a lexicon developed from the early months of the pandemic to identify key concepts within messages, employing it to analyze both the textual content of messages themselves as well as text included within embedded images (n = 233,877), which was extracted via optical character recognition. Finally, we modelled the message retransmission process using a negative binomial regression, which allowed us to quantify the extent to which particular message features amplify or suppress retransmission, net of controls related to timing and properties of the sending account. In addition to identifying other predictors of retransmission, we show that the impact of images is strongly driven by content, with textual information in messages and embedded images operating in similar ways. We offer potential recommendations for crafting and deploying social media messages that can “cut through the noise” of an infodemic.

## Introduction

Content pushed by public-facing organizations to social media could be considered as a “message in a bottle.” The “bottle” is cast out into a “sea” of communication roiled by waves of vastly divergent content, all vying for user attention; some messages arrive at their destinations while others sink. Like the bottle, not all content is created equal, either its propensity to “float” or in the importance of the enclosed message. If crucial public health messages fail to reach their intended recipients, lives could be lost. The importance of successfully communicating health messages is intensified during hazard events like an ongoing global pandemic. Public health communicators, emergency managers, and elected officials thus face the challenge of cutting through the noise of misinformation, disinformation, and competing background content to get critical messages to the public. The complexity of this information environment has been described by the World Health Organization (WHO) as an “infodemic,”^1^ encapsulating the combination of information overload, active dissemination of misinformation and unproven claims, and other messaging that compete with scientifically established facts and professional expertise. Operating within an infodemic makes the jobs of public health communicators, emergency managers, and elected officials all the more difficult.

For public communicators to cut through the noise, and successfully reach their intended audiences, it is necessary to understand what leads a message to be actively retransmitted, or shared, increasing its penetration and exposure. Here we examine several dimensions of public officials' communication strategies on Twitter during the first 8 months of the coronavirus disease 2019 (COVID-19) pandemic in the United States, from message content and structural features to the textual content of images and infographics, relating these features to observed message retransmission rates. Our analysis identified message construction tactics that could enhance retransmission in a pandemic setting, helping agencies reach members of the public within an especially challenging communication environment.

## Public Health Communication in the Pandemic Response

During times of uncertainty, individuals tend to look to officials, local or otherwise, to help make sense of what is going on around them.^2,3^ In the COVID-19 pandemic, public health and emergency management agencies and elected officials are the primary entities tasked with informing and educating the public about the state of scientific knowledge regarding virus transmission, prevention, and mitigation strategies.^4^ By bypassing intermediaries involved in traditional forms of media, social media allows for public entities to disseminate information more efficiently and to speak directly to their constituents. These organizations or officials use social media to instantaneously deliver crucial information to their audiences, often including actionable items that, if seen and heeded, can reduce health risks. Social media also allows for an active conversation with constituents, which can potentially help increase trust and credibility through open and transparent communication while correcting erroneous information that may be in circulation.^2,5^ At the same time, the factors that make social media attractive to public organizations also make it attractive to other actors, leading to an extremely crowded and ever-changing communication landscape. Navigating this shifting terrain continues to be a critical challenge.

## Information (Re)Transmission and Social Media

Twitter is widely used by officials to communicate on topics ranging from natural hazards and emergencies to issues of public health. As with other crowded social media environments, public entities posting messages on Twitter can, at best, hope to reach a very small number of users by initial, direct exposure; only a fraction of those subscribed to receive messages will be attending when a post is posted. Instead, message penetration (number of persons reached) and exposure (total views, including multiple views of the same message) are driven in large part by retransmission, where users who initially receive or otherwise find the post, pass it on to others (a process called “retweeting” on Twitter). A highly viral message may be retransmitted tens of thousands of times (resulting in extremely large numbers of exposures), while less successful messages may not be retransmitted at all. Thus, understanding the predictors of message passing is vital to effectively reaching an online audience.

Prior studies have identified several key message and sender attributes that influence message passing. These include message content and structural features, the time the message was sent, the characteristics of the sending account, and the number of followers associated with the account. Previous studies from a range of hazard contexts—including the initial weeks of the COVID-19 outbreak—have found that message content conveying the severity of threats, communicating actionable information, and including media (photos and videos) all amplify message retransmission.^6-10^ Conversely, messages that facilitated user engagement, such as direct replies and mentions, and included hyperlinks were found to lead to an attenuation of retransmission.^9,10^ One study on messaging during the first 2 months of the COVID-19 pandemic found that the use of question marks and exclamation points (both emotive but informal syntactic elements) were both associated with decreased retransmission.^10^

As noted, the use of multimedia attachments, including videos and static images, can provide significant increases in retransmission. Images, information graphics, and videos have been found to be effective tools for disseminating health-related information and helping to reach a larger audience.^11^ Infographics and data visualizations are graphic representations of information or data, such as charts or diagrams, that clearly illustrate processes, summarize key information, provide key data, or demonstrate how specific data may be interpreted.^12^ These are important elements for reaching users with limited attentional resources. Studies suggest that visual representations paired with textual information ease users' cognitive load, helping make content easier to process and remember compared to text alone.^13-16^ With public health communicators trying to increase information retention and recall, engagement, and behavioral uptake, the use of images and information graphics to communicate information has become an important tool in the public health communicator's arsenal.^17^ To date, however, the effect of image content—beyond mere inclusion—on retransmission has not been studied.

This study fills a gap in the literature on public health communication in 2 crucial ways. First, it updates our understanding of what messaging strategies are, and are not, proving effective in cutting through the noise of a pandemic, as this study considers a longer time span (8 months) than prior work in this area. Second, it establishes a quantitative evidence base for the impact of textual features embedded in images—an increasingly important tool in the COVID-19 context and in social media messaging more generally.

## Methods

### Data Collection

This study analyzed a list of 704 unique Twitter accounts, comprising public health organizations (n = 383), state governors (n = 77), state emergency management organizations (n = 50), local mayors (n = 96), and local emergency management agencies (n = 98) for the 100 largest cities in the United States. Public health accounts were identified through publicly available lists^18^ and prior projects on social media risk messaging.^6,19^ Our sampling design was purposefully constructed to ensure coverage of (1) major types of public entities involved in online COVID-19 communication, and (2) a large number of local municipalities representing a large fraction of the US population, while at the same time remaining within data collection limits. We provide a breakdown of the administration levels of these accounts as well as the complete list of sampled accounts and their associated account types in Supplemental Tables 1 and 2 (www.liebertpub.com/doi/suppl/10.1089/hs.2020.0200).

**Table 1.** Tweet Microstructural Features

Variable	Definition	Tweets* *n (%)	Example
Image	Messages coded for the presence of an image or media	185,012 (49.7)	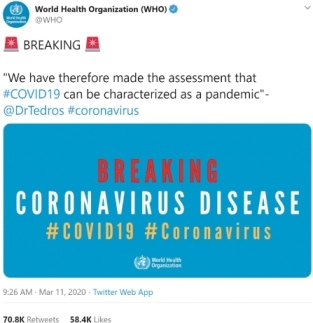
Video	Messages coded for the presence of a video	16,848 (4.5)	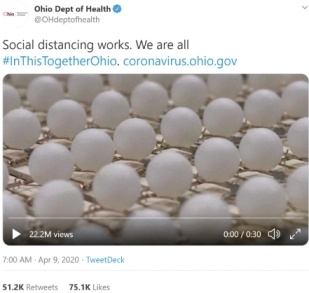
Hyperlink / URL	Message contains a hyperlink to external website	205,490 (55.0)	All Hands on Deck! Geospatial mapping meets outbreak control. To learn more about the vital role geospatial science and technology can play in public health, go to **https://t.co/lIK3Iarc9o** #CDCEHblog **https://t.co/B2lEA98OHY**
Reply	Message is in response to a tweet from another user	84,360 (22.6)	**@Cindy_Lee_G @NCCommerce** Hi, please see this link: https://t.co/vX6KfvO5Og. It includes information as well as contact information for @NCCommerce.
Mention	Message includes the Twitter handle of an individual or organization	107,308 (28.8)	Thank you **@TaosSkiValley**! #AllTogetherNM https://t.co/t0yFy9n3k8
Hashtag	Message includes a hashtag	163,595 (44.0)	Our COVID-19 site has information for businesses about how to prepare and what to do if an employee becomes sick. https://t.co/iZI0IsUjWA **#COVID19 #AZTogether**
COVID-19 Hashtags	Message contained a hashtag for coronavirus or COVID-19	74,845 (20.0) (45% of all hashtag usage)	We all can be health leaders and practice physical distancing and also wear our face coverings. **#COVID19**
Quote	Message quotes another message in its entirety	32,042 (8.6)	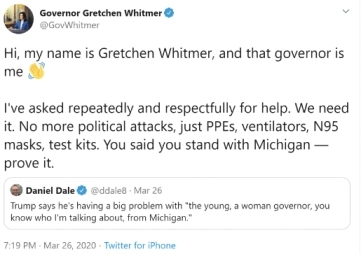
Exclamatory	Message includes an exclamation mark (!)	55,851 (14.9)	Stay Healthy Nevada**!** #StayHomeForNevada #COVID19 https://t.co/8CXK2sJcda
Interrogatory	Message includes a question mark (?)	26,272 (7.0)	Do you have questions about tenant rights and the current eviction moratorium**?** Register now for the A Way Home for Tulsa webinar on tenant rights during the COVID-19 pandemic. The webinar will be held Friday, April 3 at 9:30 a.m. #Tulsa https://t.co/IPlYOhcGEkx

We collected all 372,466 tweets produced by the 704 accounts between the dates of January 1 and August 31, 2020, using the Twitter representational state transfer (REST) application programming interface (API). This API allows for the collection of tweets by specific accounts and includes account and tweet metadata. On average, each account produced 529.1 messages during the study period, with 10 accounts producing only a single tweet and WHO producing the most (n = 6,005). A detailed list of accounts and the list of cities in the supplemental materials (www.liebertpub.com/doi/suppl/10.1089/hs.2020.0200).

#### Microstructural Features

By using regular expressions, we coded for items present within the content of the message, including microstructural features, such as structure and style. These microstructural features were coded as either present or absent and included the following features: the inclusion of a video or image, hyperlink (URL), mention (@username), direct reply, quoted tweet, hashtag, or COVID-19-specific hashtag (eg, #COVID, #Coronavirus) (Table 1).

#### Topic Lexicon Development

Lexicons are specialized keyword lists that assist in the process of identifying key topics of a specific corpus. Similar systems have been developed and leveraged in previous studies whereby researchers identified crisis-specific keywords that were relevant to an emerging disaster.^20,21^ We developed a lexicon that identifies keywords for our specific account set that (1) aligns with theoretical concepts, (2) draws on prior studies of disaster communication on Twitter, and (3) accounts for observed COVID-19 event salient content, language, and terms that appear in our corpus.

Lexical categories were constructed by manually reviewing 100 randomly sampled tweets per day from February 1 to August 31, 2020 (n* =* 21,300). We extended the lexicon of Sutton et al,^10^ which was based on the first 2 months of the COVID-19 pandemic in the United States, incorporating their content themes: closures and openings, information sharing, official information, resilience, surveillance, symptoms, and technical information. In addition to those themes, we added the category of “location” and divided the lexical category of “efficacy” into “efficacy” and “action.” Location messages contained mentions of international locations, usually in reference to places where major outbreaks of the virus had occurred, among them China, Italy, and Japan. Efficacy and action were separated based on observation of more distinct use of terms by health communicators in recent months; specifically, we found greater separation between the articulation of which protective actions to take (efficacy) and information on how to take those actions (action). It should be noted that category “action” is applicable to any action directives and is not necessarily specific to COVID-19. Further, our extension accounts for additional words or phrases that became commonplace after the month of April and are not retroactively coded. We provide the definitions of the aforementioned categories in Table 2. Additional background on the motivation for the original content categories and related coding considerations used in prior work can be found in Sutton et al.^10^

**Table 2.** Lexicon Set: Definitions, Frequencies, and Examples

Variable	Definition	In Tweets* *n (%)	In Images* *n (%)	Example (extended lexicon in italics)
Susceptibility	Keywords describing individuals or groups at risk of COVID-19	41,508 (11.0)	14,013 (7.5)	Vulnerable, risk, unlikely, travel, veteran, older, kids, age 60, chronic, immune, dialysis, diabetes, homeless, jail, shelter, facilities, African American
				*Underserved, child, immigrant, senior, unhoused, essential worker, public servant,*
Surveillance	Keywords describing strategies to identify population impact	88,380 (23.7)	33,375 (18.0)	Test, result, case, presumptive, death, contact trace, hospitalize, dashboard, sadden, recover
				*Retrace, diagnostics, intensive care, antibody test, coronavirus vaccine*
Symptoms	Keywords describing symptoms of disease	10,472 (2.8)	6,991 (3.7)	Symptom, shortness of breath, fever
Actions	Keywords instructing people on protective actions to take	92,143 (24.7)	14,466 (7.8)	Donate, follow, get tested, contact a doctor, stock up
				*Isolate, care, reengage, avoid crowds*
Efficacy	Keywords on how individuals are to take protective measures to safeguard themselves from the threat	59,735 (16.0)	22,688 (12.0)	Stay home, self-isolate, physical distance, social distance, quarantine, shelter in place, face, mask, hand wash, soap and water, 20 seconds, 6 feet, disinfect
				*Mask up, 6 feet, PPE, face covering, sanitize, cover your nose, bleach*
Collective efficacy	Keywords reflecting the capacity to achieve an intended effect	48,173 (12.9)	12,320 (6.6)	Neighbors, united, solidarity, together, community, mitigate the spread, flatten the curve, stay home save lives, shelter in place
				*Work together, crush the curve, beat the virus, move forward together*
Technical information	Keywords describing mechanism of how the virus spreads	7,973 (5.7)	13,648 (7.0)	Droplet, cough, sneeze, surface, transmission, infect, incubate, contagious
				*Talk, not showing symptoms*
Official information	Keywords about governmental responses to COVID-19 and how to access information	89,560 (24.0)	35,259 (19.0)	Public health authority, official, task force, declaration, proclamation, executive order, activate, monitor, model, advisory
				*Test site, community-based testing, testing locations, protection program, rent control*
Information sharing	Keywords that relate to outlets or events for information sharing	57,842 (15.5)	20,254 (10.0)	Helpline, briefing, livestream, broadcast, town hall, press conference, guidance
				*News release, roundtable, data-driven, based on science, public service announcement, misrepresented*
Resilience	Keywords that express thanks and appreciation	33,028 (8.8)	3,516 (10.0)	Hero, salute, thank, recognize, grateful
				*Rockstars, lit bright blue*
Closures and openings	Keywords about suspension or reinstatement of service, activities, and facilities	61,307 (16.5)	21,436 (11.0)	Suspend, close, mandatory, lockdown, visitation, cancel, large gatherings, nonessential
				*In-person, new normal, grocery, sports leagues, gym, malls*
Location	Keywords about international locales	1,151 (<1.0)	693 (<1.0)	China, Wuhan, Japan, Italy, Iran
Primary threat	Keywords used to describe COVID-19	132,392 (35.5)	52,066 (28.0)	Coronavirus, COVID-19, ncov, outbreak, pandemic
Secondary impacts	Keywords used to describe additional threats that result from the pandemic	86,859 (23.0)	24,020 (13.0)	Mental health, substance abuse, domestic violence, evict, food insecure, blood drive, scam, rumor, stigma, school, unemployment panic buy, PPE, compliance, grief

Abbreviations: COVID-19, coronavirus disease 2019; PPE, personal protective equipment.

#### Period Effects and Account Properties

We also controlled for different types of period effects and account properties (Supplemental Table 3, www.liebertpub.com/doi/suppl/10.1089/hs.2020.0200). For period effects, we controlled for the time, day of the week, and month a message was sent, and whether it was sent after the Presidential Emergency Declaration on March 15, 2020. We also controlled for the type of organization that published the message (public health agency, state emergency management, local emergency management, governor, or mayor), the log of the number of followers they have, and the log of the number of friends they have on Twitter. Specific fixed effects were added for WHO- and US Centers for Disease Control and Prevention-affiliated accounts, given their prominence and distinctive roles in the pandemic response.

**Table 3.** Negative Binomial Regression Model

	Estimate	% Change	Std. Error	P Value
Intercept	-4.962	-99.3	0.039	<.001
Account properties				
Governor account	1.273	257.2	0.009	<.001
Log follower count	0.768	115.5	0.002	<.001
Mayor account	0.532	70.2	0.008	<.001
Log(+1) friends count	-0.089	-8.5	0.002	<.001
State EM account	-0.005	-0.5	0.012	NS
Local EM account	-0.442	-35.7	0.009	<.001
CDC-affiliated accounts	0.027	2.7	0.015	NS
World Health Organization	0.158	17.1	0.023	<.001
Microstructural properties				
Incl. video	0.214	23.9	0.014	<.001
Incl. hashtag	-0.034	-3.3	0.007	<.001
Incl. image	-0.13	-12.2	0.011	<.001
Incl. quote	0.073	7.6	0.01	<.001
Incl. question mark (?)	-0.115	-10.9	0.01	<.001
Incl. mention	-0.227	-20.3	0.006	<.001
Incl. exclamation (!)	-0.19	-17.3	0.008	<.001
Incl. URL	-0.381	-31.7	0.006	<.001
Reply	-1.712	-81.9	0.007	<.001
Incl. #COVID19 hashtag	0.044	4.5	0.01	<.001
Lexical categories (message content)				
Surveillance	0.231	26.0	0.007	<.001
Technical information	0.083	8.7	0.011	<.001
Actions	-0.077	-7.4	0.006	<.001
Efficacy	0.422	52.5	0.007	<.001
Symptoms	0.127	13.5	0.016	<.001
Primary threat	0.174	19.0	0.008	<.001
Secondary impacts	0.121	12.9	0.006	<.001
Official responses	0.156	16.9	0.006	<.001
Location	0.506	65.9	0.044	<.001
Collective efficacy	0.085	8.9	0.008	<.001
Closures/openings	0.066	6.8	0.007	<.001
Resilience	-0.084	-8.1	0.009	<.001
Susceptibility	0.001	0.1	0.008	NS
Information sharing	-0.137	-12.8	0.008	<.001
Image textual content				
Image has text	0.008	0.8	0.009	N.S.
# of images	-0.018	-1.8	0.006	<.01
Surveillance	0.224	25.1	0.012	<.001
Technical information	0.054	5.5	0.016	<.001
Actions	0.032	3.3	0.015	<.05
Efficacy	0.161	17.5	0.013	<.001
Symptoms	0.122	13.0	0.021	<.001
Primary threat	0.057	5.9	0.01	<.001
Secondary impacts	0.052	5.3	0.012	<.001
Official responses	0.036	3.7	0.011	<.001
Location	0.019	1.9	0.058	NS
Collective efficacy	-0.04	-3.9	0.015	<.01
Closures/openings	0.121	12.9	0.013	<.001
Resilience	0.143	15.4	0.026	<.001
Susceptibility	0.184	20.2	0.015	<.001
Information sharing	-0.003	-0.3	0.013	NS
Period effects – national emergency declaration period				
Postdeclaration	0.256	29.2	0.014	<.001
Period effects – month				
February	-0.043	-4.2	0.014	<.01
March	0.842	132.1	0.016	<.001
April	0.381	46.4	0.018	<.001
May	0.214	23.9	0.018	<.001
June	0.411	50.8	0.018	<.001
July	0.41	50.7	0.018	<.001
August	0.23	25.9	0.018	<.001
Period effects – time of day				
12 am UTC	-0.612	-45.8	0.033	<.001
1 am UTC	-0.333	-28.3	0.034	<.001
2 am UTC	-0.131	-12.3	0.035	<.001
3 am UTC	-0.073	-7.0	0.037	NS
5 am UTC	-0.295	-25.5	0.051	<.001
6 am UTC	-0.314	-26.9	0.064	<.001
7 am UTC	0.01	1.0	0.077	NS
8 am UTC	-0.56	-42.9	0.072	<.001
9 am UTC	-0.561	-42.9	0.065	<.001
10 am UTC	-0.501	-39.4	0.049	<.001
11 am UTC	-0.668	-48.7	0.039	<.001
12 pm UTC	-0.569	-43.4	0.034	<.001
1 pm UTC	-0.583	-44.2	0.032	<.001
2 pm UTC	-0.597	-45.0	0.032	<.001
3 pm UTC	-0.656	-48.1	0.031	<.001
4 pm UTC	-0.613	-45.8	0.031	<.001
5 pm UTC	-0.617	-46.0	0.031	<.001
6 pm UTC	-0.694	-50.0	0.031	<.001
7 pm UTC	-0.567	-43.3	0.031	<.001
8 pm UTC	-0.631	-46.8	0.031	<.001
9 pm UTC	-0.597	-45.0	0.032	<.001
10 pm UTC	-0.534	-41.4	0.032	<.001
11 pm UTC	-0.379	-31.5	0.032	<.001
Period effects – day of week				
Sunday	0.318	37.4	0.011	<.001
Monday	0.099	10.4	0.009	<.001
Tuesday	0.079	8.2	0.009	<.001
Thursday	0.112	11.9	0.009	<.001
Friday	0.062	6.4	0.009	<.001
Saturday	0.139	14.9	0.01	<.001

Observations: 372,466; Akaike Information Criterion: 2,271,754

Log-likelihood: -1,1357,790; dispersion parameter: 0.514; standard error: 0.001

Note: The percent change is the exponentiated β coefficient – 1 × 100.

Abbreviations: CDC, Centers for Disease Control and Prevention; EM, emergency management; NS, not significant; UTC, coordinated universal time.

#### Optical Character Recognition

To analyze the textual content of images, we employed optical character recognition (OCR) through the use of the Image Magick and Tesseract packages for the R statistical programming language.^22-24^ OCR can convert an image that contains alphanumeric characters into a computer-readable text format.^25^ Of the 372,466 messages in our dataset, 185,012 (almost 50%) messages included an image attachment.

As part of our content coding protocol, we coded for textual information contained in images embedded in or attached to messages. To collect the images for analysis we first constructed a web scraper in R statistical programming language. This allowed us to download images for any tweets that contained an image attachment, as the link to the image is available in the tweet metadata. Since Twitter allows for the inclusion of up to 4 images in a single post (< 10% of all images contain more than 1 image), the resulting set of images to be analyzed (n = 233,877) was nearly 37 gigabytes in storage size. This set was then processed by Image Magick and analyzed by Tesseract, which took over 37 hours (approximately 1 hour per gigabyte of images on a 6-core processor). Taking all words extracted at high confidence from each image, we applied the same lexicon as used for message text to words contained within the image. The resulting content codes were then used as predictors for the message retweet rate; if multiple images were present for a given tweet, content types were merged.

Our coding was limited to static images, as videos could not be coded for semantic content. Please refer to Table 2 for descriptive information about the application of the lexical categories to the images and to Supplemental Table 4 (www.liebertpub.com/doi/suppl/10.1089/hs.2020.0200) for descriptive information about the images.

### Data Analysis

Following prior literature, we defined retransmission as the total number of times a message was retweeted. Similar to prior work on modeling retransmission on Twitter,^8^ we found that the majority of messages do not get retransmitted, with 65% being retweeted only 6 times or less, but a small fraction of messages is retweeted many thousands of times. Therefore, to estimate the contribution of mechanisms that affect the message-passing process while accounting for the heterogeneity of retransmission outcomes, we performed a negative binomial regression—using the R statistical programming language and the glmmADMB package^26,27^—on the retweet count using predictors that capture message content, style, and image textual content.

The resulting model coefficients were interpreted as follows: for a given message feature, a positive coefficient indicates an increase in message retransmission (with other conditions remaining the same), while a negative coefficient indicates a decrease in retransmission. In particular, each unit increase in a covariate increases the log of the expected retweet count by the amount of the associated coefficient. A more in-depth discussion of the negative binomial model in application to message retransmission can be found in Sutton et al.^10^

## Results

The reported model (Table 3) indicates the factors that influenced the process of retweeting the communications of public health agencies, local and state management organizations, and elected officials during the first 8 months of the COVID-19 pandemic (see Tables 1 and 2 for descriptions and frequencies of the factors). The top 3 most common content themes used by our account set (Table 2) were “actions” (n = 92,143; 25%), “official response” (n = 89,560; 24%), and “surveillance” (n = 88,380; 24%). Over 35% (n = 132,392) of messages were coded as discussing the primary threat of COVID-19 by name, and 23% (n = 86,859) of messages discussed secondary threats. In terms of message structure features (Table 1), 55% (n = 205,490) of all tweets contained a URL, 50% (n = 185,012) of all messages contained some form of image media (image, informational graphic, photograph), and 44% (n = 163,595) of all messages contained some form of hashtag, with 20% (n = 74,845) of the hashtags referencing the coronavirus. In the figures, we show predictors of retweet rates in terms of raw beta (β) coefficients. For ease of interpretation when describing results in the text below, we describe the effect of each predictor on the retweet rate in terms of the percent change in the expected retweet count associated with a unit change in the predictor—this can be obtained from the raw coefficients by subtracting 1 from the exponentiated beta coefficient and multiplying by 100.

### Message Retransmission

#### Period and Account Effects

**Figure 1.**
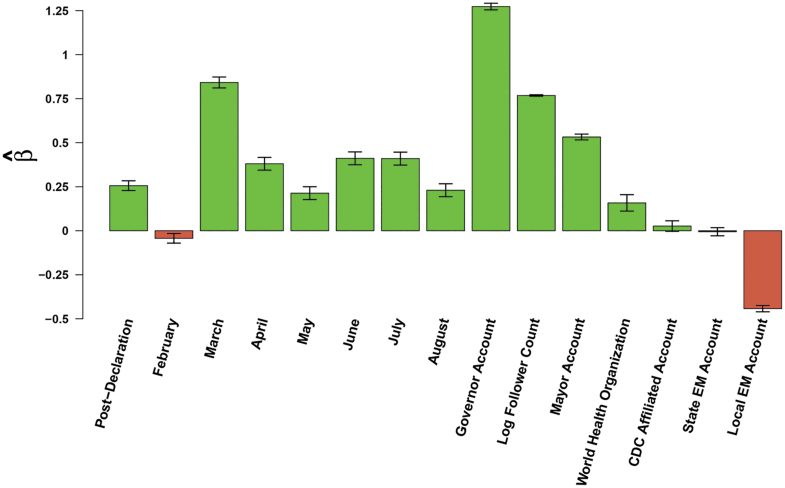
Effects of time period and account type on message retransmission. Bars indicate effects of content covariates (horizontal axis) on log expected retweet count (Table 3); whiskers indicate 95% confidence intervals.

As seen in Figure 1, the month the message was posted affected expected retransmission, suggesting overall increases or decreases in attention by the public during the 8-month period. Compared to the baseline month of January, February had a slight decrease (4%) in retransmission, followed by March, the month with the greatest focus on the coronavirus pandemic in the United States, with 132% increase to retransmission. We also found that messages posted after the emergency declaration had a 29% increase in message passing. From April to August, retransmission remained fairly steady, with slight declines in May and August.

In terms of the individual or organization posting the message, we found that with public health accounts as our baseline (the largest percentage of accounts in this dataset) that governors (257%) and mayors (70%) had the greatest impact on retransmission. Accounts associated with the US Centers for Disease Control and Prevention were not found to be significantly different than the retransmission of the public health accounts; however, the sole WHO Twitter account had a positive influence of retransmission, at 17%. Emergency management accounts at the local level had 36% lower retransmission rates, while state emergency management accounts were not significantly different from the baseline group of public health accounts. Finally, every 1 unit increase in the log follower count corresponded positively to a 116% increase in the expected retransmission rate.

#### Message Structure and Sentence Style

**Figure 2.**
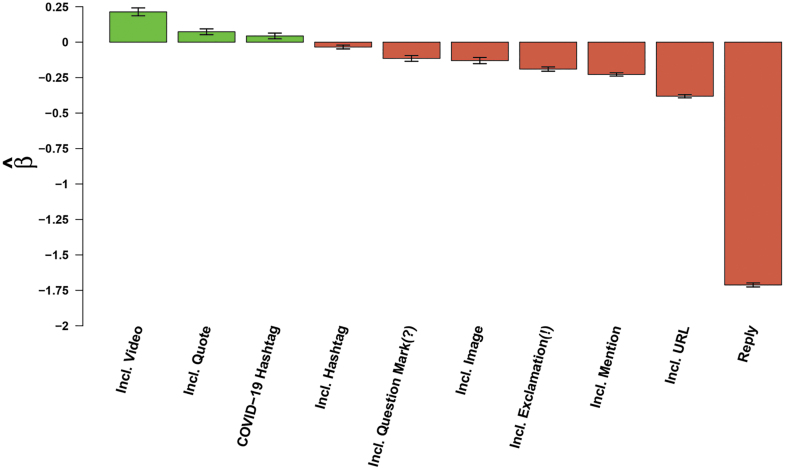
Effects of messages structural features on message retransmission. Bars indicate effects of content covariates (horizontal axis) on log expected retweet count (Table 3); whiskers indicate 95% confidence intervals.

As seen in Figure 2, we also found that message structural features can increase the potential for retransmission. Including a video in a tweet was found to increase retransmission of the associated message by 24%. Furthermore, “quote” tweets, in which a user quotes the message of another user while adding their own content, were found to increase message retransmission by 8%.

Often a large, positive influence on message retransmission, we found that the use of most hashtags negatively influenced retransmission in this context, decreasing its potential by 3%. We did, however, find that the use of #COVID19 or #Coronavirus hashtags increased retransmission by 5%, leading to a net 2% increase in retransmission overall. In contrast, inclusion of question marks or exclamation marks decreased the rate of message retransmission by 11% and 17%, respectively. Mentioning other users, which narrows conversation, and the inclusion of hyperlinks or URLs decreased message retransmission by 20% and 32%, respectively. Finally, similar to findings in studies in other contexts,^6,10^ the use of 1-to-1 responses (replies) on Twitter has a strongly negative impact on message passing, decreasing retransmission by 82%.

#### Lexical Content

**Figure 3.**
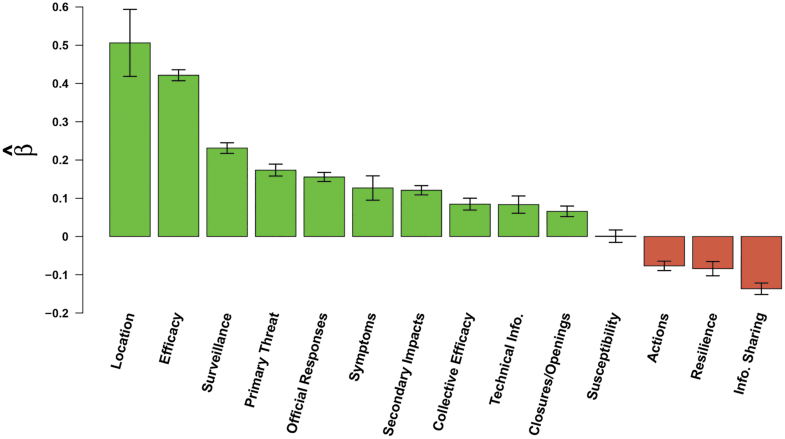
Effects of message keyword (lexical) categories on message retransmission. Bars indicate effects of content covariates (horizontal axis) on log expected retweet count (Table 3); whiskers indicate 95% confidence intervals.

The largest impact on retransmission (Figure 3) by content features is the mention of international locations associated with large COVID-19 outbreaks, corresponding to a 66% increase in retransmission, followed by efficacy (53%) and surveillance (26%) messaging. Messages that made reference to COVID-19 or coronavirus (primary threat) were found to increase retransmission by 19%. Official responses were found to increase retransmission by 17%. The mention of symptoms and secondary impacts, like mental health and other side effects of the pandemic, were each found to positively influence message retransmission by 13%. The other items that were found to positively influence message transmission were that of technical information (9%), collective efficacy (9%), and closures/openings (7%).

Three lexical categories—actions, resilience, and information sharing—were found to negatively influence the message retransmission process by 7%, 8%, and 13%, respectively. Furthermore, the model shows that messages that contained content relating to susceptibility were not found to significantly impact message retransmission in any meaningful way.

#### Image Semantic Content

**Figure 4.**
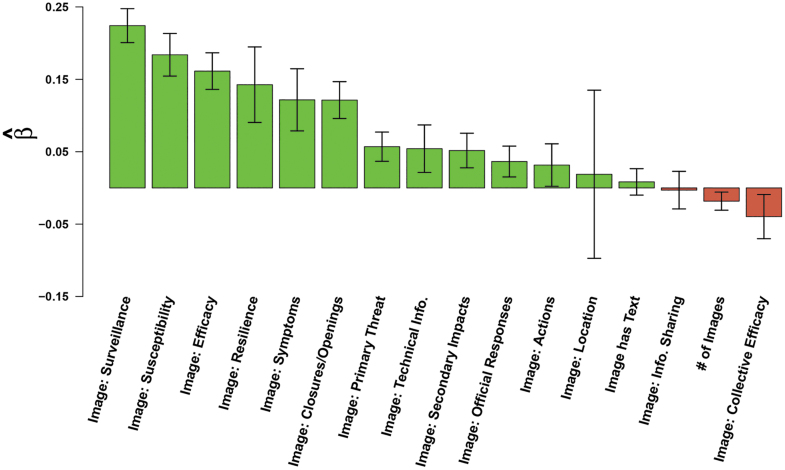
Effects of image textual content (lexical) categories on message retransmission. Bars indicate effects of content covariates (horizontal axis) on log expected retweet count (Table 3); whiskers indicate 95% confidence intervals.

Our OCR analysis (Figure 4) provided us with insights on the impact of image semantic content on message retransmission. In general, the simple inclusion of an image did not increase the potential for message retransmission—in fact, we found that including an image decreased the expected retransmission rate by 12%. An image containing generic textual content (ie, content outside the coded lexical categories) was found to have no significant impact on message retransmission, and the number of images uploaded had a weakly negative effect on message passing, with a 2% decrease per each image attached after the first image.

We also analyzed images that contained text within them. Images containing text relating to surveillance keywords, information about susceptibility, and efficacy had the greatest positive impacts on retransmission (25%, 20%, and 18%, respectively). This was followed by increased message retransmission of images with text that depict resilience (15%), discussed COVID-19-related symptoms (13%), or referenced closures and openings (13%). If image text referenced the primary threat of COVID-19 or secondary impacts associated with COVID-19, the potential for retransmission was increased by 6% and 5%, respectively. Finally, image text that had technical information (6%), discussed official responses (4%), or contained action keywords (3%) was found to positively impact retransmission. In contrast, images that conveyed information relating to collective efficacy were found to negatively impact message retransmission by 4%. The reference of international locations within an image and keywords that convey information sharing on an image did not significantly impact message retransmission. Note that all of the above effects are cumulative with both each other and with the base (negative) effect of images; thus, images without appropriate content tend to decrease retransmission rates, but images containing the right mix of content types can end up being strongly conducive to retransmission.

## Discussion

### Trends and Comparisons with Initial Outbreak Period

We found an interesting trend that could be attributed to “COVID fatigue” or “quarantine fatigue.”

Clearly, March had the most retransmissions, but by the end of April, the number of retransmissions reduced by half, and in May they nearly halved again. Although we see an uptick in retransmission that was steady in June and July, the number of retransmissions reduced again in August. March remained the most active retransmission month by far in the period studied. This trend suggests that the concept of COVID fatigue may have contributed to the post-March trends. Further research is required to confirm this hypothesis.

### Comparisons with Effects from Prior Messaging Studies

An account's organizational type has been shown to influence the message passing process.^10^ This study supports these findings. Both governor and mayor accounts were found to have higher likelihoods of retransmission, which we speculated in our previous work to potentially be a function of individuals' responsibility for decision making during the unfolding pandemic.^10^ Also, consistent with other studies,^6,9,10^ the number of followers in the immediate audience of the message increases the potential for exposure and the potential that those exposed to the message are more likely to share it with their extended network of followers.

One of the largest positive effects found in this and prior studies was the use of video media. It is apparent that within the context of information communication, the combination of video and audio leads to higher levels of message passing, compared with text-only messages, as evidenced in prior literature.^6^ We also find, in this context, that the use of quote tweets lead to a slight increase in retransmission, perhaps catching the coattails of a popular message and, thereby, expanding the initial audience for the message. However, this feature warrants further investigation to understand how the aspects of the account being quoted affect retransmission.

In line with patterns seen in other hazard contexts, this analysis also shows that there are several structural features that lead to a reduction in the potential of retransmission. Mentions and replies are some of the largest attenuators and have been found to consistently affect the message retransmission process in conditions of imminent threat,^9^ emerging infectious disease,^10^ and now over sustained durations of threat. The use of directed mentions and message replies is an important engagement strategy used by organizations, having been hypothesized to increase trust in organizations through increased levels of responsiveness.^5^ However, the same strategy also narrows the intended audience. If the content of that message could be useful to others, one should consider replying in a private message to the individual and also making a public post for a more general audience.

In agreement with prior literature,^9^ we find that the inclusion of URLs is one of the largest detractors of message passing. We believe that this is because clicking on a URL takes users away from Twitter's service, making retweeting an afterthought. Finally, the use of interrogatory language and exclamation points did not help the potential for message passing.

### Message Content and Retransmission

While style and context can matter, content is key to retransmission potential. We found that messages that discussed international locations with particularly famous outbreaks or responses (eg, Italy, South Korea, or Wuhan, China) were the most likely to be passed. This may be in part due to organizations such as WHO that discuss other countries' experiences with the coronavirus as models for treating the pandemic. Another positive factor for retransmission is efficacy, or discussion of methods of protecting oneself by limiting exposure to the threat, which was identified by keywords and phrases like “mask,” “social distancing,” and “handwash.”^6,9^ Collective efficacy was also found to help increase message passing, but to a lesser extent than efficacy. We also found tweets containing content about surveillance (eg, hospitalizations, deaths, tracing, testing) were more likely to be retransmitted, aligning with prior studies.^10^ Generally, messages containing information about hazards and their impact or severity also increased message passing.^6,9^

There were also a few lexical categories that decreased message passing, chief among them was information sharing. For instance, messaging about town halls, press conferences, and webinars did not seem to warrant retransmission since the content more likely to be passed was the video or stream itself, which had a positive retransmission effect. Finally, the categories of actions and resilience were found to attenuate message passing.

Our primary advantage over prior studies on retransmission on Twitter is our ability to analyze the semantic content of images included in a post. While past studies found that the baseline effect of merely including an image positively increased retransmission, we found that controlling for text within images unveiled that an image without text negatively influences retransmission. Therefore, for a message to be successfully retransmissible, the content, goal, approach, and strategy of the text and image or information graphic used should be considered carefully and thoughtfully. If the intent of an image or information graphic is for it to be retransmitted as widely as possible, many of the lexical categories identified here can help in that process. In addition to keywords relating to surveillance content (one of the largest promoters of retransmission), we found that susceptibility, efficacy, and resilience were the next top 3 positive effects for text in images. This differs somewhat from our results for susceptibility-themed message content, which had no influence on message retransmission, and resilience, which was found to negatively influence retransmission for message content. This could suggest that information about who is at risk (susceptibility) and information about positive concepts/terms that recognize and honor those who are risking their lives on the frontline of the pandemic (resilience) are more evocative when expressed in visual form and are overlooked when they are in purely text format. On the whole, however, we see considerable consonance between the effects of message and image content, suggesting that they may be processed in similar ways.

### Further Directions

As the pandemic evolves, it will be important to examine the impact of exogenous events that may influence retransmission rates outside the communication medium of Twitter—this might include surges/spikes of deaths or cases associated with the virus or even news associated with vaccines or other treatments. Likewise, the penetration of the virus into rural areas raises the question of whether there are distinct messaging strategies that work better (or worse) for local accounts in these regions than elsewhere. In our supplemental analysis (www.liebertpub.com/doi/suppl/10.1089/hs.2020.0200) of residual retransmission rates for local accounts, we did not find evidence that accounts associated with small cities were retweeted at different rates than larger cities, although we did see a small but significant tendency for residual retransmission rates to be slightly lower in urban areas with more than 1.5 million people. The current data do not suggest distinct effects for smaller communities, but a specialized rural sample would be required to draw more definitive conclusions. Finally, the slight increase in retransmission from quote tweets suggests value in further examining this phenomenon, in particular, whether attributes of the original poster influence the retransmission rate. Doing this type of analysis would require a specialized data collection design and could shed light on how this new platform feature could be effectively utilized.

## Recommendations for Practice

We summarize the practical implications of our findings for public health communicators, emergency management agencies, and governmental officials as follows:

- **Don't Be Cute About It:** Our findings support evidence that the public responds to and shares messages with practical information about the pandemic, its impacts, and ways to take action. Focusing on useful content rather than gimmicks to go “viral” will be helpful in the long run.
- **Use Media Critically:** Videos and images can be powerful tools to amplify messages. For images, having meaningful content embedded in them is crucial for their success—simply adding images without relevant content can potentially reduce message retransmission.
- **Not Everything Should Be an Image:** Largely, information embedded in images has a qualitatively similar impact on retransmission to information in the message text itself. Therefore, when crafting messages, focus on what information will be useful rather than through which medium it is delivered.
- **#Hashtag or Not?** Like images, hashtags are not universally useful. We found little impact with the use of COVID-19-associated hashtags and a slight negative mean impact of hashtags overall on retransmission. Past studies suggest that hashtags can be effective, but in the current pandemic they may need to be carefully targeted to have a useful effect.
- **Capitalize on Weekend Attention:** During our current pandemic, it appears that weekends are a particularly active time for message retransmission. Timing messages to hit during this period can maximize their impact.
- **Don't Narrow Your Audience:** Many of the findings from previous studies on retransmission during hazard events are also true here, including the impact of audience narrowing. While there are good reasons to use replies, mentions, and other techniques that target individuals, it is important to be aware that these approaches were found to consistently and largely reduce retransmission.
- **Cultivate Your Network (Especially Elected Officials):** While having a large follower count undoubtedly helps with retransmission, other sources of prominence also matter. High-level elected officials can direct attention in ways that local officials, public health organizations, and even some prominent government or intergovernmental agencies cannot. Allying and cooperating with these individuals to strategically boost public health messaging may be an important way to share critical information with a wide audience.

## Conclusion

During an infodemic, with misinformation and disinformation surging and swirling around us all, the use of evidence-based communication strategies is crucial. We hope that our findings will aid health communicators, emergency managers, and elected officials in crafting messages that can cut through the noise surrounding the COVID-19 pandemic.

## Supplementary Material

Supplemental data

Supplemental data

Supplemental data

Supplemental data

Supplemental data
