# Supplementary material for: Cutting Through the Noise: Predictors of Successful Online Message Retransmission in the First 8 Months of the COVID-19 Pandemic
Source: Health Secur. 2021 Feb 18;19(1):31–43. doi: 10.1089/hs.2020.0200 (PMC9195492; doi:10.1089/hs.2020.0200)
Supplement: Supplemental data [file Supp_Table1.docx]

Supplemental Table 1. Number of Accounts at Each Administrative Level

| Administration Level | Local | State | Federal | US Governmental Officials (Mayors and Governors) | International |
| --- | --- | --- | --- | --- | --- |
| Number of Accounts | 440 | 51 | 58 | 150 | 1 |
