# Supplementary material for: Cutting Through the Noise: Predictors of Successful Online Message Retransmission in the First 8 Months of the COVID-19 Pandemic
Source: Health Secur. 2021 Feb 18;19(1):31–43. doi: 10.1089/hs.2020.0200 (PMC9195492; doi:10.1089/hs.2020.0200)
Supplement: Supplemental data [file Supp_Table2.pdf]

Table 2: Organizational Names and Associated Twitter Handles

Name	Handle	Handle_2	State	City	Admin. Level	Public Health	State Emergency Management	Governor	Mayor	Local Emergency Management	Population
ZMC Health Dept	zmchd				Local	1	0	0	0	0	NA
DC Dept of Health	_dchealth				State	1	0	0	0	0	NA
Northampton Health	01060health				Local	1	0	0	0	0	NA
Anne Arundel Health	aahealth				Local	1	0	0	0	0	NA
Ashland-Boyd Co. HD	abchdky				Local	1	0	0	0	0	NA
Alamance Health Dept	achealthdept				Local	1	0	0	0	0	NA
Nantucket Health Dpt	ackhealthdept				Local	1	0	0	0	0	NA
AllenCoPublicHealth	acpublichealth				Local	1	0	0	0	0	NA
Ark. Dept. of Health	adhpio				State	1	0	0	0	0	NA
Alaska DHSS	alaska_dhss				State	1	0	0	0	0	NA
Allegan County HD	allegancountyhd				Local	1	0	0	0	0	NA
Allegany Co Health	alleganyhlthmd				Local	1	0	0	0	0	NA
Ala. Public Health	alpublichealth				State	1	0	0	0	0	NA
AppDistrictHD	appdistricthd				Local	1	0	0	0	0	NA
Austin Public Health	auspublichealth				Local	1	0	0	0	0	NA
Avon Board of Health	avonboh				Local	1	0	0	0	0	NA
AZ Dept. of Health Verified account	azdhs				State	1	0	0	0	0	NA
Barren River Health	barrenriverhd				Local	1	0	0	0	0	NA
Beach Cities Health	bchd				Local	1	0	0	0	0	NA
Brown County Health	bchd_georgetown				Local	1	0	0	0	0	NA
Barnstable Co Health	bchdcapecod				Local	1	0	0	0	0	NA
Bear River Health	bearriverhealth				Local	1	0	0	0	0	NA
BethlehemHealthDept	bethlehemhealth				Local	1	0	0	0	0	NA
BFHD	bfhd				Local	1	0	0	0	0	NA
BHC Health Dept	bhchd				Local	1	0	0	0	0	NA
B'more City Health Verified account	bmore_healthy				Local	1	0	0	0	0	NA
Boone County Health	boonecohealth				Local	1	0	0	0	0	NA
Bo Co Public Health	bouldercohealth				Local	1	0	0	0	0	NA
Brant Health Unit	branthealthunit				Local	1	0	0	0	0	NA
CDC's Behavioral Risk Factor Surveillance System	brfss				Federal	1	0	0	0	0	NA
Brown Co Health Dept	browncohealth				Local	1	0	0	0	0	NA
Butler County Health	butlercounty_hd				Local	1	0	0	0	0	NA
BwD Public Health	bwdph				Local	1	0	0	0	0	NA
Cal. Co. Health Dept	calverthealth				Local	1	0	0	0	0	NA
CAPublicHealth	capublichealth				State	1	0	0	0	0	NA
CarverCoPublicHealth	carvercoph				Local	1	0	0	0	0	NA
CCBH_Net	ccbh_net				Local	1	0	0	0	0	NA
Clinton Co MO HD	cchdmissouri				Local	1	0	0	0	0	NA
Clark Co Health Dept	cchealthdept				Local	1	0	0	0	0	NA
Carson City Health	cchealthed				Local	1	0	0	0	0	NA
CCPHD	ccphd				Local	1	0	0	0	0	NA
	cdc_cancer				Federal	1	0	0	0	0	NA
CDC_eHealth Verified account	cdc_ehealth				Federal	1	0	0	0	0	NA
	cdc_eidjournal				Federal	1	0	0	0	0	NA
	cdc_ephtracking				Federal	1	0	0	0	0	NA
	cdc_hivaids				Federal	1	0	0	0	0	NA
NCBDDD Verified account	cdc_ncbddd				Federal	1	0	0	0	0	NA
	cdc_ncbddd				Federal	1	0	0	0	0	NA
	cdc_ncezid				Federal	1	0	0	0	0	NA
	cdc_tb				Federal	1	0	0	0	0	NA
	cdcchronic				Federal	1	0	0	0	0	NA
Dr. Tom Frieden Verified account	cdcdirector				Federal	1	0	0	0	0	NA
CDC Emergency Verified account	cdcemergency				Federal	1	0	0	0	0	NA
	cdcenvironment				Federal	1	0	0	0	0	NA
	cdcespanol				Federal	1	0	0	0	0	NA
CDC Flu Verified account	cdcflu				Federal	1	0	0	0	0	NA
FluGov Verified account	cdcflu				Federal	1	0	0	0	0	NA
	cdcglobal				Federal	1	0	0	0	0	NA
CDC Verified account	cdcgov				Federal	1	0	0	0	0	NA
	cdchep				Federal	1	0	0	0	0	NA
	cdcinjury				Federal	1	0	0	0	0	NA
	cdckenya				Federal	1	0	0	0	0	NA
	cdcmakehealthez				Federal	1	0	0	0	0	NA
MMWR Verified account	cdcmmwr				Federal	1	0	0	0	0	NA
CDC NPIN Verified account	cdcnpin				Federal	1	0	0	0	0	NA
	cdcobesity				Federal	1	0	0	0	0	NA
	cdcsouthafrica				Federal	1	0	0	0	0	NA
	cdcstd				Federal	1	0	0	0	0	NA
CDC Tobacco Free Verified account	cdctobaccofree				Federal	1	0	0	0	0	NA
	cdctravel				Federal	1	0	0	0	0	NA
CDPHE	cdphe				State	1	0	0	0	0	NA
C&D Public Health	cdphga				Local	1	0	0	0	0	NA
Cecil County Health	cecilcohealth				Local	1	0	0	0	0	NA
CGC Dept. of Health	cgpublichealth				Local	1	0	0	0	0	NA
Public Health	chelandouglashd				Local	1	0	0	0	0	NA

Chesco Health Verified account	chescohealth	Local	1	0	0	0	0	NA
ChiPublicHealth Verified account	chipublichealth	Local	1	0	0	0	0	NA
CinciHealthDept	cincihealthdept	Local	1	0	0	0	0	NA
CityofSTLDOH	cityofstldoh	Local	1	0	0	0	0	NA
CCCHD	clarkcohealth	Local	1	0	0	0	0	NA
Clark County WA_PH	clarkcowa_ph	Local	1	0	0	0	0	NA
Clay Co. Pub. Health	claycountymn	Local	1	0	0	0	0	NA
Clay Co Health Dept Verified account	claymohealth	Local	1	0	0	0	0	NA
CDPH	cledph	Local	1	0	0	0	0	NA
ClintonCoHealthDept	clintoncohealth	Local	1	0	0	0	0	NA
Contra Costa Health Verified account	cocohealth	Local	1	0	0	0	0	NA
CDPHE EPR	cohealth	State	1	0	0	0	0	NA
Columbus Health	columbushealth	Local	1	0	0	0	0	NA
Columbia/BooneHealth	como_healthdept	Local	1	0	0	0	0	NA
CCDPH	cookcohealth	Local	1	0	0	0	0	NA
Champaign Health	cpublichealth	Local	1	0	0	0	0	NA
CU Public Health	cpublichealth	Local	1	0	0	0	0	NA
CraterHealthDistrict	craterhd	Local	1	0	0	0	0	NA
CenRacineCoHealth	crchd	Local	1	0	0	0	0	NA
CT Public Health	ctdph	State	1	0	0	0	0	NA
CU Public Health	cu_publichealth	Local	1	0	0	0	0	NA
CUPHD	cuphd	Local	1	0	0	0	0	NA
Darke County Health	darkecohealth	Local	1	0	0	0	0	NA
Davis County Health	daviscountyhlth	Local	1	0	0	0	0	NA
Defiance Co. Health	dcghd	Local	1	0	0	0	0	NA
Del.Co Public Health	dcphs	Local	1	0	0	0	0	NA
	debhourycdc	Federal	1	0	0	0	0	NA
DHSS	delaware_dhss	State	1	0	0	0	0	NA
Delaware Health Dist	delawarehealth	Local	1	0	0	0	0	NA
Denver Public Health	denpublichealth	Local	1	0	0	0	0	NA
Detroit Health Dept	dethealth	Local	1	0	0	0	0	NA
WIDeptHealthServices	dhswi	State	1	0	0	0	0	NA
	drkhabbazcdc	Federal	1	0	0	0	0	NA
	drmartincdc	Federal	1	0	0	0	0	NA
	drmermincdc	Federal	1	0	0	0	0	NA
DCHD	dupagehd	Local	1	0	0	0	0	NA
Durham Health	durhamhealth	Local	1	0	0	0	0	NA
Durham Public Health	durhamhealthnc	Local	1	0	0	0	0	NA
ECPHD	eastcentralph	Local	1	0	0	0	0	NA
Erie Cty Health Dept	ecdoh	Local	1	0	0	0	0	NA
EnglewoodHealthDept	englewoodhealth	Local	1	0	0	0	0	NA
Public Health	epcpublichealth	Local	1	0	0	0	0	NA
EP Public Health	eppublichealth	Local	1	0	0	0	0	NA
FairfaxCounty Health Verified account	fairfaxhealth	Local	1	0	0	0	0	NA
Franklin County PH	fc_publichealth	Local	1	0	0	0	0	NA
FC Health Department	fchealthdept	Local	1	0	0	0	0	NA
FCPublicHealth	fcph	Local	1	0	0	0	0	NA
FDA Drug Information Verified account	fda_drug_info	Federal	1	0	0	0	0	NA
U.S. FDA Verified account	fdarecalls	Federal	1	0	0	0	0	NA
FDA Tobacco Verified account	fdatobacco	Federal	1	0	0	0	0	NA
FEMA Verified account	fema	Federal	1	0	0	0	0	NA
Broward Health Dept	flhealthbroward	Local	1	0	0	0	0	NA
FSRIO at NAL Verified account	foodsafety	Federal	1	0	0	0	0	NA
foodsafety.gov Verified account	foodsafetygov	Federal	1	0	0	0	0	NA
Health & Human Ser.	fortbendhealth	Local	1	0	0	0	0	NA
Fulton Health	fultonhealth	Local	1	0	0	0	0	NA
Gallatin Health Dept	gallatinhealth	Local	1	0	0	0	0	NA
Gaston HHS	gastonhhs	Local	1	0	0	0	0	NA
GF Public Health	gfpublichealth	Local	1	0	0	0	0	NA
GNRHealthDepartments	gnrhealth	Local	1	0	0	0	0	NA
Geauga County LHD	gphohio	Local	1	0	0	0	0	NA
Green River Health	grdhd	Local	1	0	0	0	0	NA
GreendaleHD	greendalehd	Local	1	0	0	0	0	NA
GC Public Health	greenecoph	Local	1	0	0	0	0	NA
Greene NY Health	greenenyhealth	Local	1	0	0	0	0	NA
Greenup Co Health	greenuphealth	Local	1	0	0	0	0	NA
Grinnell Regional	grmc	Local	1	0	0	0	0	NA
GTCounty Health Dept	gtchd	Local	1	0	0	0	0	NA
Hamilton Co. Health	hamcohealth	Local	1	0	0	0	0	NA
ChattHamiltonHealth	hamiltonhealth	Local	1	0	0	0	0	NA
HarCo Health Dept.	harcohealthdept	Local	1	0	0	0	0	NA
Hartford HHS	hartfordhealth	Local	1	0	0	0	0	NA
HCHPH	hchph	Local	1	0	0	0	0	NA
Health District	healthdistrict	Local	1	0	0	0	0	NA
DOUGLASCOUNTYHEALTH	healthdouglasco	Local	1	0	0	0	0	NA
NYSDOH Verified account	healthnygov	State	1	0	0	0	0	NA
VT Dept of Health	healthvermont	State	1	0	0	0	0	NA
Wayne County Health	healthwayne	Local	1	0	0	0	0	NA
Williams Co. Health	healthwchd	Local	1	0	0	0	0	NA

Boston Public Health	healthyboston	Local	1	0	0	0	0	NA
DeKalb County BOH	healthydekalb	Local	1	0	0	0	0	NA
Florida Dept. Health	healthyfla	State	1	0	0	0	0	NA
Hull Board of Health	healthyhull	Local	1	0	0	0	0	NA
Mo Health & Sr Srvcs Verified account	healthylivingmo	State	1	0	0	0	0	NA
Mason County Health	healthymc	Local	1	0	0	0	0	NA
OK Dept of Health	healthyoklahoma	State	1	0	0	0	0	NA
HendricksHealthDept	hendricks_co	Local	1	0	0	0	0	NA
Henry County HD	henrycountyhd	Local	1	0	0	0	0	NA
HHS.gov Verified account	hhsgov	Federal	1	0	0	0	0	NA
Hidalgo Co. Health	hidalgohealth	Local	1	0	0	0	0	NA
HI Dept of Health	higov_health	State	1	0	0	0	0	NA
AIDS.gov Verified account	hivgov	Federal	1	0	0	0	0	NA
Howard Co Health Dep Verified account	hocohealth	Local	1	0	0	0	0	NA
Houston Health Dept	houstonhealth	Local	1	0	0	0	0	NA
Humboldt County DHHS Verified account	humcodhhs	Local	1	0	0	0	0	NA
HCPH	huroncohealth	Local	1	0	0	0	0	NA
IDPH - Public Health	iapublichealth	State	1	0	0	0	0	NA
ICPHD	icpublichealth	Local	1	0	0	0	0	NA
IDHW	idhw	State	1	0	0	0	0	NA
IDPH	idph	State	1	0	0	0	0	NA
IDPH-OPR	idph	State	1	0	0	0	0	NA
Mobile County Health	ilovemchd	Local	1	0	0	0	0	NA
Ingham Health Dept.	inghamhealth	Local	1	0	0	0	0	NA
OWH	inwomenshealth	State	1	0	0	0	0	NA
JessCo Health Dept.	jchd_ky	Local	1	0	0	0	0	NA
JCPHS	jcphs	Local	1	0	0	0	0	NA
JeffCoHealth	jeffcohealthmo	Local	1	0	0	0	0	NA
Jeffco Public Health Verified account	jeffcoph	Local	1	0	0	0	0	NA
JCDHE Verified account	jocohealth	Local	1	0	0	0	0	NA
JoCo Public Health	jocoph	Local	1	0	0	0	0	NA
Kane County Health	kanecohealth	Local	1	0	0	0	0	NA
KCMO Health Dept	kcmohealthdept	Local	1	0	0	0	0	NA
Public Health - SKC	kcpubhealth	Local	1	0	0	0	0	NA
KDHE	kdhe	State	1	0	0	0	0	NA
Knox Co Health Dept	knoxcohealthtn	Local	1	0	0	0	0	NA
Knox County Health	knoxhealth	Local	1	0	0	0	0	NA
Ky. Public Health	kyhealthalerts	State	1	0	0	0	0	NA
La Dept. of Health	ladepthealth	State	1	0	0	0	0	NA
Lake Co. Health Dept	lakecohealth	Local	1	0	0	0	0	NA
LA Public Health	lapublichealth	Local	1	0	0	0	0	NA
Larimer Health Dept.	larimerhealth	Local	1	0	0	0	0	NA
Long Beach Health	lbhealthdept	Local	1	0	0	0	0	NA
Health Department	lcdhd	Local	1	0	0	0	0	NA
LCHC	lchcia	Local	1	0	0	0	0	NA
Lake Cty Health Dist	lchealthdist	Local	1	0	0	0	0	NA
Linn Public Health	lcpublichealth	Local	1	0	0	0	0	NA
LDCHealth	ldchealth	Local	1	0	0	0	0	NA
Ledge Light HD	ledgelighthd	Local	1	0	0	0	0	NA
Lenawee Health Dept.	lenaweehd	Local	1	0	0	0	0	NA
LexKy Health Dept.	lfchd	Local	1	0	0	0	0	NA
Licking Co. Health	lickinghealth	Local	1	0	0	0	0	NA
Lorain County Health	loraincohealth	Local	1	0	0	0	0	NA
LouMetroHealth	loumetrohealth	Local	1	0	0	0	0	NA
Macoupin County PHD	macoupinhealth	Local	1	0	0	0	0	NA
MadisonCountyHealth	madisonchd	Local	1	0	0	0	0	NA
Madison Co HD, KY	madisoncountyhd	Local	1	0	0	0	0	NA
Public Health	maricopahealth	Local	1	0	0	0	0	NA
Public Health	maricopahealth	Local	1	0	0	0	0	NA
MarinHealth&HumanSvc Verified account	marinhhs	Local	1	0	0	0	0	NA
Marion Co. Health	marion_health	Local	1	0	0	0	0	NA
Marion County Health	marionco_health	Local	1	0	0	0	0	NA
Mass. Public Health	massdph	State	1	0	0	0	0	NA
Healthy Mesa County	mc_publichealth	Local	1	0	0	0	0	NA
MCHD & Public Health	mchd_tx	Local	1	0	0	0	0	NA
Monterey Co Health	mchdpio	Local	1	0	0	0	0	NA
McHenryCountyHealth	mchenrycohealth	Local	1	0	0	0	0	NA
McLeanCountyHealth	mcleanhealth	Local	1	0	0	0	0	NA
DHMH Verified account	mdhealthdept	State	1	0	0	0	0	NA
Health Department	meckcounty	Local	1	0	0	0	0	NA
Health Department	meckcounty	Local	1	0	0	0	0	NA
Medina County Health	medinahealth	Local	1	0	0	0	0	NA
Maine CDC	mepublichealth	State	1	0	0	0	0	NA
Miami County Health	miamicountyph	Local	1	0	0	0	0	NA
Michigan HHS Dept Verified account	michiganhhs	State	1	0	0	0	0	NA
	millionheartsus	Federal	1	0	0	0	0	NA
Milton BOH	miltonboh	Local	1	0	0	0	0	NA
Ottawa County Health	miocdph	Local	1	0	0	0	0	NA
MKE Health Dept.	mkehealth	Local	1	0	0	0	0	NA
MDH	mnhealth	State	1	0	0	0	0	NA
Montgomery CountyHHS	mocodhhs	Local	1	0	0	0	0	NA
Monroe County Health	monroecountyhea	Local	1	0	0	0	0	NA

Monroe County Health	monroehealth	Local	1	0	0	0	0	NA
MS Dept of Health	msdh	State	1	0	0	0	0	NA
Missoula Health Dept	mslahealthdept	Local	1	0	0	0	0	NA
MultCoHealth	multcohealth	Local	1	0	0	0	0	NA
NashvilleHealth	nashvillehealth	Local	1	0	0	0	0	NA
NC DHHS Verified account	ncdhhs	State	1	0	0	0	0	NA
N.CentralHealthDist.	nchd52	Local	1	0	0	0	0	NA
	nchstats	Federal	1	0	0	0	0	NA
NC DPH	ncpublichealth	State	1	0	0	0	0	NA
N.D. Dept. of Health	nddoh	State	1	0	0	0	0	NA
Nebraska DHHS	nedhhs	State	1	0	0	0	0	NA
Needham PublicHealth	needham_health	Local	1	0	0	0	0	NA
NET Health	nethealthfits	Local	1	0	0	0	0	NA
Newton Health & HS	newtonhealth	Local	1	0	0	0	0	NA
North Georgia Health	ngahealthdist	Local	1	0	0	0	0	NA
DHHS PIO	nhdhhspio	State	1	0	0	0	0	NA
NewHaven Health Dept	nhpublichealth	Local	1	0	0	0	0	NA
NIH Verified account	nih	Federal	1	0	0	0	0	NA
NIH NHLBI Verified account	nih_nhlbi	Federal	1	0	0	0	0	NA
Mental Health NIMH Verified account	nimhgov	Federal	1	0	0	0	0	NA
NIOSH Verified account	niosh	Federal	1	0	0	0	0	NA
	niosh_twh	Federal	1	0	0	0	0	NA
	nioshconstruct	Federal	1	0	0	0	0	NA
	nioshespanol	Federal	1	0	0	0	0	NA
	nioshface	Federal	1	0	0	0	0	NA
	nioshmining	Federal	1	0	0	0	0	NA
	nioshnoise	Federal	1	0	0	0	0	NA
NJDOH	njdeptofhealth	State	1	0	0	0	0	NA
NKY Health Dept	nkyhealth	Local	1	0	0	0	0	NA
NM Dept. of Health	nmdoh	State	1	0	0	0	0	NA
Northeast CO Health	northeasthealth	Local	1	0	0	0	0	NA
Norwalk Health Dept.	norwalkhealth	Local	1	0	0	0	0	NA
Norwood Health Dept	norwoodhd	Local	1	0	0	0	0	NA
	niosh_npptl	Federal	1	0	0	0	0	NA
NWCOVNA	nwcolohealth	Local	1	0	0	0	0	NA
nwhealth.org	nwhealthorg	Local	1	0	0	0	0	NA
Oak Creek Health	oakcreekhealth	Local	1	0	0	0	0	NA
Health Care Agency	ochealth	Local	1	0	0	0	0	NA
Onslow County Health	ochealthdept	Local	1	0	0	0	0	NA
Health Department	ocpublichealth	Local	1	0	0	0	0	NA
OR Health Authority	ohaoregon	State	1	0	0	0	0	NA
Ohio Dept of Health	ohdeptofhealth	State	1	0	0	0	0	NA
OCCHD	okchealth	Local	1	0	0	0	0	NA
Orange County Health	orangehealthnc	Local	1	0	0	0	0	NA
St. Mary's Health	oswegohealth	Local	1	0	0	0	0	NA
Pennsylvania DOH Verified account	pahealthdept	State	1	0	0	0	0	NA
Pima County Health	pchd	Local	1	0	0	0	0	NA
Peoria Hlth Dept IL	peoriaprepare	Local	1	0	0	0	0	NA
PequannockTwp HD Gov	peqtwp_health	Local	1	0	0	0	0	NA
PGC Health Dept.	pgchealth	Local	1	0	0	0	0	NA
Panhandle Health Verified account	phdidaho	Local	1	0	0	0	0	NA
Idaho Health Dist. 2	phincd	Local	1	0	0	0	0	NA
Idaho Health Dist. 2	phincd	Local	1	0	0	0	0	NA
Philly Public Health Verified account	phlpublichealth	Local	1	0	0	0	0	NA
PHS District Health	phshealthdept	Local	1	0	0	0	0	NA
PikeCountyHealthDep	pikecountyhd	Local	1	0	0	0	0	NA
Platte County Health	plattehealth	Local	1	0	0	0	0	NA
Polk County Health	polkcohealth	Local	1	0	0	0	0	NA
Polk Co Health Ctr	polkcountyhc	Local	1	0	0	0	0	NA
Preble County Health	preblecohealth	Local	1	0	0	0	0	NA
PCCHU Health Unit	ptbohealth	Local	1	0	0	0	0	NA
Barron Public Health	publichealthbc	Local	1	0	0	0	0	NA
Public Health DMC	publichealthdmc	Local	1	0	0	0	0	NA
publichealthOC	publichealthoc	Local	1	0	0	0	0	NA
PuebloHealthDept	pueblohealth	Local	1	0	0	0	0	NA
Putnam County Health	putnamcohealth	Local	1	0	0	0	0	NA
PutnamHealthDeptNY	putnamhealthny	Local	1	0	0	0	0	NA
Readygov Verified account	readygov	Federal	1	0	0	0	0	NA
Richland Health	richlandhealth1	Local	1	0	0	0	0	NA
RI Dept. of Health	rihealth	State	1	0	0	0	0	NA
Rockland County DOH	rockhealth	Local	1	0	0	0	0	NA
Ross County Health	rosscohealth	Local	1	0	0	0	0	NA
Sac County DHHS	sacpublichealth	Local	1	0	0	0	0	NA
Sac County DHHS	sacpublichealth	Local	1	0	0	0	0	NA
Salem BoardOfHealth	salemhealthdept	Local	1	0	0	0	0	NA
Salt Lake Co. Health Verified account	saltlakehealth	Local	1	0	0	0	0	NA
SA Metro Health	sametrohealth	Local	1	0	0	0	0	NA
SAMHSA Verified account	samhsagov	Federal	1	0	0	0	0	NA
Sandusky County HD	sanduskycohd	Local	1	0	0	0	0	NA
Sauk Co Health Dept	saukcohealth	Local	1	0	0	0	0	NA
SBC Public Health	sbcpublichealth	Local	1	0	0	0	0	NA

St Charles Co Health	scchealth	Local	1	0	0	0	0	NA
St.Croix Pub Health	sccwiess	Local	1	0	0	0	0	NA
SCDHEC	scdhec	State	1	0	0	0	0	NA
South Central Health	schd_5_1	Local	1	0	0	0	0	NA
SC Dept of Health	schealthdept	Local	1	0	0	0	0	NA
SchuylerPublicHealth	schuylercoph	Local	1	0	0	0	0	NA
SummitCoPublicHealth	scphoh	Local	1	0	0	0	0	NA
SD HHSA Verified account	sdcountyhhsa	Local	1	0	0	0	0	NA
Department of Health Verified account	sddoh	State	1	0	0	0	0	NA
SEU Health District	seuthealth	Local	1	0	0	0	0	NA
SFDPH	sf_dph	Local	1	0	0	0	0	NA
S-GC Health Dept.	sgchd	Local	1	0	0	0	0	NA
Shelby County Health Verified account	shelbytnhealth	Local	1	0	0	0	0	NA
SIPH	siphidaho	Local	1	0	0	0	0	NA
SLO Public Health Verified account	slopublichealth	Local	1	0	0	0	0	NA
St. Mary's Health	smchd_gov	Local	1	0	0	0	0	NA
SMC Health System	smchealth	Local	1	0	0	0	0	NA
SMDHU	smdhealthunit	Local	1	0	0	0	0	NA
SN Health District	snhdflu	Local	1	0	0	0	0	NA
SN Health District Verified account	snhdinfo	Local	1	0	0	0	0	NA
SnoHD	snohd	Local	1	0	0	0	0	NA
SE Health District	southhealthdist	Local	1	0	0	0	0	NA
SRHD	spokanehealth	Local	1	0	0	0	0	NA
Indiana Health Dept	statehealthin	State	1	0	0	0	0	NA
StClairCounty Health	stclairhealth	Local	1	0	0	0	0	NA
St. Louis County DPH	stlcountydoh	Local	1	0	0	0	0	NA
StoneCountyHealthDp	stonecohealth	Local	1	0	0	0	0	NA
Sullivan Co. Health	sullivanhealth	Local	1	0	0	0	0	NA
SW Public Health Dep	swpublichealth	Local	1	0	0	0	0	NA
Taney Co Health Dept	taneycohealth	Local	1	0	0	0	0	NA
TCHD Emergency Verified account	tchdhealth	Local	1	0	0	0	0	NA
Tri-County Health Verified account	tchdhealth	Local	1	0	0	0	0	NA
Texas DSHS	texasdshs	State	1	0	0	0	0	NA
ThurstonHealth	thurstonhealth	Local	1	0	0	0	0	NA
TN Dept. of Health	tndeptofhealth	State	1	0	0	0	0	NA
Toledo Lucas	toledolucashd	Local	1	0	0	0	0	NA
TooeleCoHealthDept	tooelehealth	Local	1	0	0	0	0	NA
Amherst Health Dept	townofamherst	Local	1	0	0	0	0	NA
Concord MA Pub Hlth	townofconcordma	Local	1	0	0	0	0	NA
Pierce County Health	tpchd	Local	1	0	0	0	0	NA
3 Rivers Health Dept	trdhd	Local	1	0	0	0	0	NA
TulareCo HHSA	tulareco_hhsa	Local	1	0	0	0	0	NA
Tulsa Health Dept	tulsahealthdept	Local	1	0	0	0	0	NA
TuSNHD	tusnhd	Local	1	0	0	0	0	NA
UtahCountyHealthDept	uchd	Local	1	0	0	0	0	NA
Patrick McCormack	uncashealth	Local	1	0	0	0	0	NA
Dept. of Agriculture Verified account	usda	Federal	1	0	0	0	0	NA
Utah Dept. of Health Verified account	utahdepofhealth	State	1	0	0	0	0	NA
Health Care Agency	vcpublichealth	Local	1	0	0	0	0	NA
Va Dept of Health	vdhgov	State	1	0	0	0	0	NA
VintonCo Health Dept	vintoncohd	Local	1	0	0	0	0	NA
Vineland Health Dept	vldhealth	Local	1	0	0	0	0	NA
WacoMc Public Health	wacohealthdept	Local	1	0	0	0	0	NA
WA Dept. of Health	wadepthealth	State	1	0	0	0	0	NA
WCCHD	warrencohealth	Local	1	0	0	0	0	NA
Wasatch County HD	wasatchcountyhd	Local	1	0	0	0	0	NA
MontereyCoPubHealth	washcoverstay	Local	1	0	0	0	0	NA
Wash. Co. HD of Ohio	washingtoncooh	Local	1	0	0	0	0	NA
Washoe County Health	washoehealth	Local	1	0	0	0	0	NA
OZ Public Health	washozph	Local	1	0	0	0	0	NA
WCCHealth District	wcchd	Local	1	0	0	0	0	NA
Wood County Health	wchdohio	Local	1	0	0	0	0	NA
Warren County Health	wchealth	Local	1	0	0	0	0	NA
Westchester Health	wchealthdept	Local	1	0	0	0	0	NA
WC Public Health	wcpublichealth	Local	1	0	0	0	0	NA
Weber-Morgan Health	webermorganhd	Local	1	0	0	0	0	NA
Webster Co. Health	websterpublic	Local	1	0	0	0	0	NA
WhatcomCountyHealth	whatcomcohealth	Local	1	0	0	0	0	NA
World Health Organization	who	International	1	0	0	0	0	NA
Wicomico Health	wicomicohealth	Local	1	0	0	0	0	NA
Will County Health	willcohealth	Local	1	0	0	0	0	NA
WCCHealth District	williamsontn	Local	1	0	0	0	0	NA
WinnCoHealth	winncohealth	Local	1	0	0	0	0	NA
Winnebago Co Health	winnebagohealth	Local	1	0	0	0	0	NA
Winona Health	winonahealth	Local	1	0	0	0	0	NA
Western NSW LHD	wnswlhd	Local	1	0	0	0	0	NA
WDPH	worcesterdph	Local	1	0	0	0	0	NA
Worcester Health	worcesterhealth	Local	1	0	0	0	0	NA

Wright County PH	wrightcountymn			Local	1	0	0	0	0	NA
W.Springfield Health	wspfdhealthdept			Local	1	0	0	0	0	NA
WV DHHR	wv_dhhr			State	1	0	0	0	0	NA
WW Community Health	wwcdch			Local	1	0	0	0	0	NA
Yakima Health Dist.	yakimahealth			Local	1	0	0	0	0	NA
Alabama	alabamaema		Alabama	Local	0	1	0	0	0	NA
Alaska	alaskadhsem		Alaska	Local	0	1	0	0	0	NA
Arizona	aznationalguard		Arizona	Local	0	1	0	0	0	NA
Arkansas	ar_emergencies		Arkansas	Local	0	1	0	0	0	NA
California	cal_oes		California	Local	0	1	0	0	0	NA
Colorado	coemergency		Colorado	Local	0	1	0	0	0	NA
Connecticut	ctdemhs		Connecticut	Local	0	1	0	0	0	NA
Delaware	delawareema		Delaware	Local	0	1	0	0	0	NA
Florida	flsert		Florida	Local	0	1	0	0	0	NA
Georgia	georgiaema		Georgia	Local	0	1	0	0	0	NA
Hawaii	hawaii_ema		Hawaii	Local	0	1	0	0	0	NA
Idaho	idahooem		Idaho	Local	0	1	0	0	0	NA
Illinois	readyillinois		Illinois	Local	0	1	0	0	0	NA
Indiana	idhs		Indiana	Local	0	1	0	0	0	NA
Iowa	iowahsemd		Iowa	Local	0	1	0	0	0	NA
Kansas	kansasemergency		Kansas	Local	0	1	0	0	0	NA
Kentucky	kentuckyem		Kentucky	Local	0	1	0	0	0	NA
Louisiana	gohsep		Louisiana	Local	0	1	0	0	0	NA
Maine	maineema		Maine	Local	0	1	0	0	0	NA
Maryland	mdmema		Maryland	Local	0	1	0	0	0	NA
Massachusetts	massema		Massachusetts	Local	0	1	0	0	0	NA
Michigan	michemhs		Michigan	Local	0	1	0	0	0	NA
Minnessota	mndps_hsem		Minnessota	Local	0	1	0	0	0	NA
Mississippi	msema		Mississippi	Local	0	1	0	0	0	NA
Missouri	mosema_		Missouri	Local	0	1	0	0	0	NA
Montana	montanades		Montana	Local	0	1	0	0	0	NA
Nebraska	nematweets		Nebraska	Local	0	1	0	0	0	NA
Nevada	nvemergencymgmt		Nevada	Local	0	1	0	0	0	NA
New Hampshire	nh_hsem		New Hampshire	Local	0	1	0	0	0	NA
New Jersey	njohsp		New Jersey	Local	0	1	0	0	0	NA
New Mexico	nmdhsem		New Mexico	Local	0	1	0	0	0	NA
New York	nysdhses		New York	Local	0	1	0	0	0	NA
North Carolina	ncemergency		North Carolina	Local	0	1	0	0	0	NA
North Dakota	nddes		North Dakota	Local	0	1	0	0	0	NA
Ohio	ohio_ema		Ohio	Local	0	1	0	0	0	NA
Oklahoma	okem		Oklahoma	Local	0	1	0	0	0	NA
Oregon	oregonoem		Oregon	Local	0	1	0	0	0	NA
Pennsylvania	pemahq		Pennsylvania	Local	0	1	0	0	0	NA
Rhode Island	rhodeislandema		Rhode Island	Local	0	1	0	0	0	NA
South Carolina	scemd		South Carolina	Local	0	1	0	0	0	NA
South Dakota	sdemergencymgmt		South Dakota	Local	0	1	0	0	0	NA
Tennessee	t_e_m_a		Tennessee	Local	0	1	0	0	0	NA
Texas	tdem		Texas	Local	0	1	0	0	0	NA
Utah	utahdps		Utah	Local	0	1	0	0	0	NA
Vermont	vemvt		Vermont	Local	0	1	0	0	0	NA
Virginia	vdem		Virginia	Local	0	1	0	0	0	NA
Washington	waemd		Washington	Local	0	1	0	0	0	NA
West Virginia	wvdhsem		West Virginia	Local	0	1	0	0	0	NA
Wisconsin	readywisconsin		Wisconsin	Local	0	1	0	0	0	NA
Wyoming	wyohs		Wyoming	Local	0	1	0	0	0	NA
Kay Ivey	governorkayivey		Alabama	Governor	0	0	1	0	0	NA
Mike Dunleavy	govdunleavy		Alaska	Governor	0	0	1	0	0	NA
Doug Ducey	dougducey		Arizona	Governor	0	0	1	0	0	NA
Asa Hutchinson	asahutchinson		Arkanasas	Governor	0	0	1	0	0	NA
Gavin Newsom	gavinnewsom		California	Governor	0	0	1	0	0	NA
Jared Polis	govofco		Colorado	Governor	0	0	1	0	0	NA
Ned Lamont	govnedlamont		Connecticut	Governor	0	0	1	0	0	NA
John Carney	johncarneyde		Delaware	Governor	0	0	1	0	0	NA
Ron DeSantis	govrondesantis		Florida	Governor	0	0	1	0	0	NA
Brian Kemp	govkemp		Georgia	Governor	0	0	1	0	0	NA
Lou Leon Guerrero	louleonguerrero		Guam	Governor	0	0	1	0	0	NA
David Ige	govhawaii		Hawaii	Governor	0	0	1	0	0	NA
Brad Little	governorlittle		Idaho	Governor	0	0	1	0	0	NA
JB Pritzker	govpritzker	jbpritzker	Illinois	Governor	0	0	1	0	0	NA
Eric Holcomb	govholcomb		Indiana	Governor	0	0	1	0	0	NA
Kim Reynolds	iagovernor	kimreynoldsia	Iowa	Governor	0	0	1	0	0	NA

Laura Kelly	govlaurakelly	laurakellyks	Kansas		Governor	0	0	1	0	0	NA
Andy Beshear	govandybeshear	andybeshearky	Kentucky		Governor	0	0	1	0	0	NA
John Bel Edwards	louisianagov	johnbelforla	Louisiana		Governor	0	0	1	0	0	NA
Janet Mills	govjanetmills		Maine		Governor	0	0	1	0	0	NA
Larry Hogan	govlarryhogan	larryhogan	Maryland		Governor	0	0	1	0	0	NA
Charlie Baker	massgovernor	charliebakerma	Massachusetts		Governor	0	0	1	0	0	NA
Gretchen Whitmer	govwhitmer	gretchenwhitmer	Michigan		Governor	0	0	1	0	0	NA
Tim Walz	govtimwalz	tim_walz	Minnesot		Governor	0	0	1	0	0	NA
Tate Reeves	tatereeves		Mississippi		Governor	0	0	1	0	0	NA
Mike Parson	govparsonmo	mikeparson	Missouri		Governor	0	0	1	0	0	NA
Steve Bullock	governorbullock	stevebullockmt	Montana		Governor	0	0	1	0	0	NA
Pete Ricketts	govricketts		Nebraska		Governor	0	0	1	0	0	NA
Steve Sisolak	govsisolak	stevesisolak	Nevada		Governor	0	0	1	0	0	NA
Chris Sununu	govchrissununu	chrissununu	New Hampshire		Governor	0	0	1	0	0	NA
Phil Murphy	govmurphy	philmurphynj	New Jersey		Governor	0	0	1	0	0	NA
Michelle Lujan Grisham	govmlg		New Mexico		Governor	0	0	1	0	0	NA
Andrew Cuomo	nygovcuomo		New York		Governor	0	0	1	0	0	NA
Roy Cooper	nc_governor	roycoopernc	North Carolina		Governor	0	0	1	0	0	NA
Doug Burgum	dougburgum	dougfordakota	North Dakota		Governor	0	0	1	0	0	NA
Mike DeWine	govmikedewine	mikedewine	Ohio		Governor	0	0	1	0	0	NA
Kevin Stitt	govstitt		Oklahoma		Governor	0	0	1	0	0	NA
Kate Brown	oregongovbrown		Oregon		Governor	0	0	1	0	0	NA
Tom Wolf	governortomwolf		Pennsylvania		Governor	0	0	1	0	0	NA
Wanda Vazquez Garced	wandavazquezg		Puerto Rico		Governor	0	0	1	0	0	NA
Gina Raimondo	govraimondo	ginaraimondo	Rhode Island		Governor	0	0	1	0	0	NA
Henry McMaster	henrymcmaster		South Carolina		Governor	0	0	1	0	0	NA
Kristi Noem	govkristinoem	kristinoem	South Dakota		Governor	0	0	1	0	0	NA
Bill Lee	govbilllee		Tennessee		Governor	0	0	1	0	0	NA
Greg Abbott	govabbott	gregabbott_tx	Texas		Governor	0	0	1	0	0	NA
Gary R. Herbert	govherbert		Utah		Governor	0	0	1	0	0	NA
Phil Scott	govphilscott		Vermont		Governor	0	0	1	0	0	NA
Albert Bryan Jr	govbryan		Virgin Islands		Governor	0	0	1	0	0	NA
Ralph Northam	governorva		Virginia		Governor	0	0	1	0	0	NA
Jay Inslee	govinslee	jayinslee	Washington		Governor	0	0	1	0	0	NA
Jim Justice	wvgovernor	jimjusticewv	West Virginia		Governor	0	0	1	0	0	NA
Tony Evers	govevers	tony4wi	Wisconsin		Governor	0	0	1	0	0	NA
Mark Gordon	governorgordon		Wyoming		Governor	0	0	1	0	0	NA
Bill de Blasio	nycmayor	nycmayorsoffice	New York	New York City	Mayor	0	0	0	1	0	8336817
Eric Garcetti	mayorofla		California	Los Angeles	Mayor	0	0	0	1	0	3979576
Lori Lightfoot	chicagosmayor		Illinois	Chicago City	Mayor	0	0	0	1	0	2693976
Sylvester Turner	sylvesterturner		Texas	Houston	Mayor	0	0	0	1	0	2320268
Kate Gallego	mayorgallego		Arizona	Phoenix	Mayor	0	0	0	1	0	1680992
Jim Kenney	phillymayor		Pennsylvania	Philadelphia	Mayor	0	0	0	1	0	1584064
Ron Nirenberg	ron_nirenberg		Texas	San Antonio	Mayor	0	0	0	1	0	1547253
Kevin Faulconer	kevin_faulconer	sdmayorsoffice	California	San Diego	Mayor	0	0	0	1	0	1423851
Eric Johnson	johnson4dallas	dallasmayor	Texas	Dallas	Mayor	0	0	0	1	0	1343573
Sam Liccardo	sliccardo		California	San Jose	Mayor	0	0	0	1	0	1021795
Steve Adler	mayoradler		Texas	Austin	Mayor	0	0	0	1	0	978908
Lenny Curry	lennycurry		Florida	Jacksonville	Mayor	0	0	0	1	0	911507
Betsy Price	mayorbetsyprice		Texas	Fort Worth	Mayor	0	0	0	1	0	909585
Andrew Ginther	mayorginther		Ohio	Columbus	Mayor	0	0	0	1	0	898553

London Breed	londonbreed		California	San Francisco	Mayor	0	0	0	1	0	881549
Vi Lyles	cltmayor		North Carolina	Charlotte	Mayor	0	0	0	1	0	885708
Joe Hogsett	indymayorjoe		Indiana	Indianapolis	Mayor	0	0	0	1	0	876384
Jenny Durkan	mayorjenny	officeofmayor	Washington	Seattle	Mayor	0	0	0	1	0	753675
Michael Hancock	mayorhancock		Colorado	Denver	Mayor	0	0	0	1	0	727211
Muriel Bowser	mayorbowser		District of Columbia	Washington	Mayor	0	0	0	1	0	705749
Marty Walsh	marty_walsh		Massachusetts	Boston	Mayor	0	0	0	1	0	692600
Dee Margo	mayor_margo		Texas	El Paso	Mayor	0	0	0	1	0	681728
Mike Duggan	mayormikeduggan		Michigan	Detroit	Mayor	0	0	0	1	0	670031
John Cooper	johncooper4nash		Tennessee	Nashville	Mayor	0	0	0	1	0	670820
Ted Wheeler	tedwheeler		Oregon	Portland	Mayor	0	0	0	1	0	654741
Jim Strickland	mayormemphis		Tennessee	Memphis	Mayor	0	0	0	1	0	651073
David Holt	davidfholt		Oklahoma	Oklahoma City	Mayor	0	0	0	1	0	655057
Carolyn Goodman	mayoroflasvegas		Nevada	Las Vegas	Mayor	0	0	0	1	0	651319
Greg Fischer	louisvillemayor		Kentucky	Louisville	Mayor	0	0	0	1	0	617638
Bernard C. Jack Young	mayorbcyoung		Maryland	Baltimore	Mayor	0	0	0	1	0	593490
Tom Barrett	mayorofmke		Wisconsin	Milwaukee City	Mayor	0	0	0	1	0	590157
Tim Keller	mayorkeller		New Mexico	Albuquerque	Mayor	0	0	0	1	0	560513
Regina Romero	tucsonromero		Arizona	Tucson	Mayor	0	0	0	1	0	548073
Lee Brand	mayorleebrand		California	Fresno	Mayor	0	0	0	1	0	531576
John Giles	mayorgiles		Arizona	Mesa	Mayor	0	0	0	1	0	518012
Darrell Steinburg	mayor_steinberg		California	Sacramento	Mayor	0	0	0	1	0	513624
Keisha Lance Bottoms	keishabottoms		Georgia	Atlanta	Mayor	0	0	0	1	0	506811
Quinton Lucas	mayorlucaskc		Missouri	Kansas City	Mayor	0	0	0	1	0	495327
John Suthers	johnsuthers	mayorofcos	Colorado	Colorado Springs	Mayor	0	0	0	1	0	478221
Francis X. Suarez	francissuarez		Florida	Miami City	Mayor	0	0	0	1	0	467963
Mary-Ann Baldwin	maryannbaldwin		North Carolina	Raleigh	Mayor	0	0	0	1	0	474069
Jean Stothert	jean_stothert		Nebraska	Omaha	Mayor	0	0	0	1	0	478192
Robert Garcia	longbeachmayor		California	Long Beach	Mayor	0	0	0	1	0	462628
Bobby Dyer	bobbydyervb		Virginia	Virginia Beach	Mayor	0	0	0	1	0	449974
Libby Schaaf	libbyschaaf		California	Oakland	Mayor	0	0	0	1	0	433031
Jacob Frey	mayorfrey	jacob_frey	Minnesota	Minneapolis	Mayor	0	0	0	1	0	429606
G. T. Bynum	gtbynum		Oklahoma	Tulsa	Mayor	0	0	0	1	0	401190
Jeff Williams	mayorjwilliams		Texas	Arlington	Mayor	0	0	0	1	0	398854
Jane Castor	janecastor		Florida	Tampa	Mayor	0	0	0	1	0	399700
LaToya Cantrell	mayorcantrell		Louisiana	New Orleans	Mayor	0	0	0	1	0	390144
Dr. Brandon Whipple	bwhippleks		Kansas	Wichita	Mayor	0	0	0	1	0	389938
Karen Goh	karenkgoh		California	Bakersfield	Mayor	0	0	0	1	0	384145
Harry Sidhu	mayorharrysidhu		California	Anaheim	Mayor	0	0	0	1	0	350365
Kirk Caldwell	mayorkirkhnl		Hawaii	Honolulu	Mayor	0	0	0	1	0	345064
Miguel Pulido	mayorpulido		California	Santa Ana	Mayor	0	0	0	1	0	332318
Rusty Bailey	mayor_bailey		California	Riverside	Mayor	0	0	0	1	0	331360
Linda Gorton	mayorgorton		Kentucky	Lexington	Mayor	0	0	0	1	0	323152
Michael Tubbs	michaeldtubbs		California	Stockton	Mayor	0	0	0	1	0	312697
Debra March	debra_march		Nevada	Henderson	Mayor	0	0	0	1	0	320189
Melvin Carter	mayorcarter		Minnesota	St. Paul City	Mayor	0	0	0	1	0	308096
Lyda Krewson	lydakrewson		Missouri	St. Louis	Mayor	0	0	0	1	0	300576
john Cranley	johncranley		Ohio	Cincinnati	Mayor	0	0	0	1	0	303940
Bill Peduto	billpeduto	thenextpgh	Pennsylvania	Pittsburgh	Mayor	0	0	0	1	0	300286
Nancy B. Vaughan	vaughannancy		North Carolina	Greensboro	Mayor	0	0	0	1	0	296710
Ethan Berkowitz	mayorethananc		Alaska	Anchorage	Mayor	0	0	0	1	0	288000
Harry LaRosiliere	planomayorharry		Texas	Plano	Mayor	0	0	0	1	0	287677
Leirion Gaylor Baird	mayorleirion		Nebraska	Lincoln	Mayor	0	0	0	1	0	289102
Buddy Dyer	orlandomayor		Florida	Orlando	Mayor	0	0	0	1	0	287442
Christina L. Shea	sheaonirvine		California	Irvine	Mayor	0	0	0	1	0	287401
Ras J. Baraka	rasjbaraka		New Jersey	Newark	Mayor	0	0	0	1	0	282011
Wade Kapszukiewicz	wadekaps		Ohio	Toledo	Mayor	0	0	0	1	0	272779
Steve Schewel	stevefordurham		North Carolina	Durham	Mayor	0	0	0	1	0	278993
Mary Casilla Salas	mayormarysalas		California	Chula Vista	Mayor	0	0	0	1	0	274492
Tom Henry	mayortomhenry		Indiana	Fort Wayne	Mayor	0	0	0	1	0	270402
Steven Fulop	stevenfulop		New Jersey	Jersey City	Mayor	0	0	0	1	0	262075
Rick Kriseman	kriseman		Florida	St. Petersburg	Mayor	0	0	0	1	0	265351
Pete Saenz	mayorpetesaenz		Texas	Laredo	Mayor	0	0	0	1	0	262491
Satya Rhodes-Conway	mayorofmadison		Wisconsin	Madison	Mayor	0	0	0	1	0	259680
Kevin Hartke	chandlerazmayor		Arizona	Chandler	Mayor	0	0	0	1	0	261165
Byron W. Brown	byronwbrown		New York	Buffalo	Mayor	0	0	0	1	0	255284
Dan Pope	mayordanpope		Texas	Lubbock	Mayor	0	0	0	1	0	258862
Jim Lane	mayorjimlane		Arizona	Scottsdale	Mayor	0	0	0	1	0	258069
Hillary Schieve	mayorschieve		Nevada	Reno	Mayor	0	0	0	1	0	255601
Jerry Weiers	mayorweiers		Arizona	Glendale	Mayor	0	0	0	1	0	252381
Jenn Daniels	gilbertazmayor		Arizona	Gilbert	Mayor	0	0	0	1	0	254114
John Lee	mayorjohnlee		Nevada	North Las Vegas	Mayor	0	0	0	1	0	251974
Kenneth Alexander	kennyalexander		Virginia	Norfolk	Mayor	0	0	0	1	0	242742
Carolos Hernandez	mayorhialeah		Florida	Hialeah	Mayor	0	0	0	1	0	233339
Lily Mei	lilymei4fremont		California	Fremont	Mayor	0	0	0	1	0	241110
Lauren McLean	laurenmclean		Idaho	Boise	Mayor	0	0	0	1	0	228959
Levar Stoney	levarstoney		Virginia	Richmond	Mayor	0	0	0	1	0	230436
Sharon Weston Broome	mayorbroome		Louisiana	Baton Rouge	Mayor	0	0	0	1	0	220236
David Condon	david_a_condon		Washington	Spokane	Mayor	0	0	0	1	0	222081
Michael S. Purzycki	mikepurzycki		Delaware	Wilmington	Mayor	0	0	0	1	0	70166
Miro Weinberger	btvmayor		Vermont	Burlington	Mayor	0	0	0	1	0	42819
Amy Goodwin	amysgoodwin		West Virginia	Charleston	Mayor	0	0	0	1	0	46536
Marian J. Orr	gofishwyo		Wyoming	Cheyenne	Mayor	0	0	0	1	0	64235
NYC Emergency Management	nycemergencymgt		New York	New York City	Local	0	0	0	0	1	8336817
Emergency Management Department	readyla		California	Los Angeles	Local	0	0	0	0	1	3979576

Office of Emergency Management & Communications	chicagooemc	notifychicago	Illinois	Chicago City	Local	0	0	0	0	1	2693976
Office of Emergency Management	houstonoem	alerthouston	Texas	Houston	Local	0	0	0	0	1	2320268
Office of Homeland Security & Emergency Management	maricopaemerg		Arizona	Phoenix	Local	0	0	0	0	1	1680992
Office of Emergency Management	philaoem		Pennsylvania	Philadelphia	Local	0	0	0	0	1	1584064
Office of Emergency Management	sanantoniooem		Texas	San Antonio	Local	0	0	0	0	1	1547253
Office of Emergency Services	readysandiego		California	San Diego	Local	0	0	0	0	1	1423851
Office of Emergency Management	dallasoem		Texas	Dallas	Local	0	0	0	0	1	1343573
Office of Emergency Management	cityofsanjose	scc_oes	California	San Jose	Local	0	0	0	0	1	1021795
Homeland Security and Emergency Management	austinhsem		Texas	Austin	Local	0	0	0	0	1	978908
City of Jacksonville Emergency Preparedness	jaxready		Florida	Jacksonville	Local	0	0	0	0	1	911507
Emergency Management Office	fwoem		Texas	Fort Worth	Local	0	0	0	0	1	909585
The Office of Emergency Preparedness	cphoep		Ohio	Columbus	Local	0	0	0	0	1	898553
Department of Emergency Management	sf_emergency		California	San Francisco	Local	0	0	0	0	1	881549
Charlotte-Mecklenburg Emergency Management	charmeckem	preppete_cmemo	North Carolina	Charlotte	Local	0	0	0	0	1	885708
Indianapolis Office of Public Health and Safety	indyophs		Indiana	Indianapolis	Local	0	0	0	0	1	876384
Office of Emergency Management	oemseattle		Washington	Seattle	Local	0	0	0	0	1	753675
Office of Emergency Management	denveroem		Colorado	Denver	Local	0	0	0	0	1	727211
Homeland Security and Emergency Management Agency	dc_hsema		District of Columbia	Washington	Local	0	0	0	0	1	705749
Emergency Management	alertboston		Massachusetts	Boston	Local	0	0	0	0	1	692600
Office of Emergency Management	elpasooem		Texas	El Paso	Local	0	0	0	0	1	681728
Homeland Security and Emergency Management	cityofdetroit		Michigan	Detroit	Local	0	0	0	0	1	670031
Office of Emergency Management	nashvilleeoc		Tennessee	Nashville	Local	0	0	0	0	1	670820
Portland Bureau of Emergency Management	portlandbem		Oregon	Portland	Local	0	0	0	0	1	654741
Office of Emergency Management	memphisoem		Tennessee	Memphis	Local	0	0	0	0	1	651073
Office of Emergency Management	cityofokc		Oklahoma	Oklahoma City	Local	0	0	0	0	1	655057
Emergency Management	clvalerts		Nevada	Las Vegas	Local	0	0	0	0	1	651319
Emergency Management Agency	loumetroes		Kentucky	Louisville	Local	0	0	0	0	1	617638
Mayor's Office of Emergency Management	baltimoreoem		Maryland	Baltimore	Local	0	0	0	0	1	593490
Office of Emergency Management	readymke		Wisconsin	Milwaukee City	Local	0	0	0	0	1	590157
Office of Emergency Management	abq_oem		New Mexico	Albuquerque	Local	0	0	0	0	1	560513
Office of Emergency Management & Homeland Security	toemhs		Arizona	Tucson	Local	0	0	0	0	1	548073
Office of Emergency Services	fresnooes		California	Fresno	Local	0	0	0	0	1	531576
Maricopa County Department of Emergency Management	maricopaemerg		Arizona	Mesa	Local	0	0	0	0	1	518012
Office of Emergency Management	sacramentooes		California	Sacramento	Local	0	0	0	0	1	513624
Office of Emergency Preparedness	atlpreparedness		Georgia	Atlanta	Local	0	0	0	0	1	506811
Office of Emergency Management	kcmooem		Missouri	Kansas City	Local	0	0	0	0	1	495327
Pikes Peek Regional Office of Emergency Management	cityofcos		Colorado	Colorado Springs	Local	0	0	0	0	1	478221
Miami-Dade County Office of Emergency Management	miamidadeem		Florida	Miami City	Local	0	0	0	0	1	467963
Emergency Management	wakegov		North Carolina	Raleigh	Local	0	0	0	0	1	474069
Douglas County Emergency Management	dcema_nebraska		Nebraska	Omaha	Local	0	0	0	0	1	478192
Disaster Preparedness	lbdisasterprep		California	Long Beach	Local	0	0	0	0	1	462628
Office of Emergency Management	cityofvabeach		Virginia	Virginia Beach	Local	0	0	0	0	1	449974

Emergency Management Services Division	oakland911	California	Oakland	Local	0	0	0	0	1	433031
Hennepin County Emergency Management	hennepinem	Minnesota	Minneapolis	Local	0	0	0	0	1	429606
Tulsa Area Emergency Management Agency	cityoftulsagov	Oklahoma	Tulsa	Local	0	0	0	0	1	401190
Office of Emergency Management	cityofarlington	Texas	Arlington	Local	0	0	0	0	1	398854
Office of Emergency Management	alerttampa	Florida	Tampa	Local	0	0	0	0	1	399700
Office of Homeland Security & Emergency Preparedness	nolaready	Louisiana	New Orleans	Local	0	0	0	0	1	390144
Sedgwick County Emergency Management	sedgwickcounty	Kansas	Wichita	Local	0	0	0	0	1	389938
Emergency Management /Cuyahoga County	cuyahogaoem	Ohio	Cleveland City	Local	0	0	0	0	1	381009
Kern County Fire Department's Office of Emergency Services	kerncountyfire	California	Bakersfield	Local	0	0	0	0	1	384145
Office of Emergency Management/Adams County Emergency Management	adamsoem	Colorado	Aurora	Local	0	0	0	0	1	379289
Emergency Management & Preparedness/Orange County, CA	ocsheriff	California	Anaheim	Local	0	0	0	0	1	350365
Department of Emergency Management	oahu_dem	Hawaii	Honolulu	Local	0	0	0	0	1	345064
Emergency Management/Orange County, CA	ocsheriff	California	Santa Ana	Local	0	0	0	0	1	332318
Emergency Management Department	rivcoready	California	Riverside	Local	0	0	0	0	1	331360
Office of Emergency Management	readycc	Texas	Corpus Christi	Local	0	0	0	0	1	326586
Emergency Management	lexkyem	Kentucky	Lexington	Local	0	0	0	0	1	323152
Office of Emergency Services	stocktonupdates	California	Stockton	Local	0	0	0	0	1	312697
Emergency Management	readystpaul	Minnesota	St. Paul City	Local	0	0	0	0	1	308096
Emergency Management Agency	cityema	Missouri	St. Louis	Local	0	0	0	0	1	300576
Hamilton County Emergency Management Agency and Homeland Security	hcema	Ohio	Cincinnati	Local	0	0	0	0	1	303940
Office of Emergency Management & Homeland Security	pittsburghoemhs	Pennsylvania	Pittsburgh	Local	0	0	0	0	1	300286
Emergency Management	greensborocity	North Carolina	Greensboro	Local	0	0	0	0	1	296710
Office of Emergency Management	anchorage_oem	Alaska	Anchorage	Local	0	0	0	0	1	288000
Emergency Management	planoem	Texas	Plano	Local	0	0	0	0	1	287677
Lancaster County Emergency Management	lancastercoema	Nebraska	Lincoln	Local	0	0	0	0	1	289102
Emergency Management	citybeautiful	Florida	Orlando	Local	0	0	0	0	1	287442
Office of Emergency Management/Orange County, CA	ocsheriff	California	Irvine	Local	0	0	0	0	1	287401
Emergency Management	cityofnewarknj	New Jersey	Newark	Local	0	0	0	0	1	282011
Lucas County Emergency Management Agency	lucascoema	Ohio	Toledo	Local	0	0	0	0	1	272779
Emergency Management Department	alertdurham	North Carolina	Durham	Local	0	0	0	0	1	278993
Fire Department's Department of Emergency Management	chulavistafd	California	Chula Vista	Local	0	0	0	0	1	274492
Allen County Department of Health's Emergency Preparedness	allencountyinfo	Indiana	Fort Wayne	Local	0	0	0	0	1	270402
Hudson County Office of Emergency Management	hudconjoem	New Jersey	Jersey City	Local	0	0	0	0	1	262075
Pinellas County Emergency Management	pinellasem	Florida	St. Petersburg	Local	0	0	0	0	1	265351
Dane County Emergency Management	danecountyem	Wisconsin	Madison	Local	0	0	0	0	1	259680
Maricopa County Department of Emergency Management	maricopaemerg	Arizona	Chandler	Local	0	0	0	0	1	261165
Erie County Emergency Management Civil Defense/Homeland Security Division	eriecountyesu	New York	Buffalo	Local	0	0	0	0	1	255284
Office of Emergency Management	cityoflubbock	Texas	Lubbock	Local	0	0	0	0	1	258862
Office of Emergency Management	scottsdaleazgov	Arizona	Scottsdale	Local	0	0	0	0	1	258069

Washoe County Emergency Management	washoecounty	covid19washoe	Nevada	Reno	Local	0	0	0	0	1	255601
Emergency Management	glendaleaz		Arizona	Glendale	Local	0	0	0	0	1	252381
Emergency Management	gilbertyourtown		Arizona	Gilbert	Local	0	0	0	0	1	254114
Forsyth County Emergency Management	readyforsyth		North Carolina	Winston-Salem	Local	0	0	0	0	1	247945
Emergency Management	cnlv		Nevada	North Las Vegas	Local	0	0	0	0	1	251974
Department of Emergency Management	norfolkdepr		Virginia	Norfolk	Local	0	0	0	0	1	242742
Fire Department's Emergency Management	chesapeakefire		Virginia	Chesapeake	Local	0	0	0	0	1	244835
Office of Emergency Management	thecityofirving		Texas	Irving	Local	0	0	0	0	1	239798
Office of Emergency Services	fremont_ca		California	Fremont	Local	0	0	0	0	1	241110
Ada County Emergency Management & Community Resillience	adacountyem		Idaho	Boise	Local	0	0	0	0	1	228959
Office of Emergency Management	rfdva		Virginia	Richmond	Local	0	0	0	0	1	230436
Mayor's Office of Homeland Security Emergency Preparedness	redstickready		Louisiana	Baton Rouge	Local	0	0	0	0	1	220236
Greater Spokane Emergency Management	gegemergencymgt		Washington	Spokane	Local	0	0	0	0	1	222081
Office of Emergency Management	cityofwilmde		Delaware	Wilmington	Local	0	0	0	0	1	70166
Office of Emergency Management/cumberland county emergency management	cityportland	ccemaready	Maine	Portland	Local	0	0	0	0	1	66215
Homeland Security and Emergency Management/kanawha county em	kanawhaus		West Virginia	Charleston	Local	0	0	0	0	1	46536
Laramie County Emergency Management	cityoflaramie		Wyoming	Cheyenne	Local	0	0	0	0	1	64235
