# Supplementary material for: Cutting Through the Noise: Predictors of Successful Online Message Retransmission in the First 8 Months of the COVID-19 Pandemic
Source: Health Secur. 2021 Feb 18;19(1):31–43. doi: 10.1089/hs.2020.0200 (PMC9195492; doi:10.1089/hs.2020.0200)
Supplement: Supplemental data [file Supp_Table3.docx]

Supplemental Table 3. Account and Period Effects: Descriptive Information

| Account/Period Type | Description | Frequency | % of Total Tweets |
| --- | --- | --- | --- |
| Public Health |  | 183,497 | 49% |
| *Elected Official* |  |  |  |
| Governor | Governor accounts from all 50 states; D.C. and P.R. | 57,758 | 15.5% |
| Mayor | Mayors of top 100 most populated cities | 67,660 | 18% |
| *Emergency Management* |  |  |  |
| State | State EM accounts from all 50 states; D.C. and P.R. | 21,042 | 5.6% |
| Local | Local EM accounts of top 100 most populated cities. | 42,509 | 11% |
| Period Effects |  |  |  |
| *Months* |  |  |  |
| January |  | 26,697 | 7% |
| February |  | 27,342 | 7% |
| March |  | 59,096 | 15.8% |
| April |  | 62,977 | 17% |
| May |  | 53,689 | 14% |
| June |  | 46,932 | 12.6% |
| July |  | 48,830 | 13% |
| August |  | 46,903 | 12.6% |
| *National Emergency Declaration* |  |  |  |
| Before |  | 70,464 | 19% |
| After |  | 302,002 | 81% |
