# Supplementary material for: Cutting Through the Noise: Predictors of Successful Online Message Retransmission in the First 8 Months of the COVID-19 Pandemic
Source: Health Secur. 2021 Feb 18;19(1):31–43. doi: 10.1089/hs.2020.0200 (PMC9195492; doi:10.1089/hs.2020.0200)
Supplement: Supplemental data [file Supp_Table4.docx]

Supplemental Table 4. Image Specific Features: Definitions, Descriptive Information, and Examples

| Image Feature | Definition | Frequency | % of Tweets | % of Images |
| --- | --- | --- | --- | --- |
| *# of Images* | How many images were included > 1. | 18,538 | 5% | 10% |
| 2 Images | Contained 2 images. | 9,583 | 2.5% | 5% |
| 3 Images | Contained 3 images. | 4,430 | 1% | 2% |
| 4 Images | Contained 4 images. | 4,525 | 1% | 2% |
| Images with text | Images that contained text. | 124,497 | 33% | 67% |
