# Supplementary material for: Cutting Through the Noise: Predictors of Successful Online Message Retransmission in the First 8 Months of the COVID-19 Pandemic
Source: Health Secur. 2021 Feb 18;19(1):31–43. doi: 10.1089/hs.2020.0200 (PMC9195492; doi:10.1089/hs.2020.0200)
Supplement: Supplemental data [file Supp_Data.docx]

**Supplemental Analysis**

*Extended Discussion of Frequency Table*

Supplemental Table 4 (www.liebertpub.com/doi/suppl/10.1089/hs.2020.0200) showcases the content categories found in the text embedded in images attached to tweets within this 8-month period. As seen in the previous table, 50% of all messages contained an image; of those tweets that contained an image, 67% contained some form of textual content (*n* = 124,497). 18,538 tweets contained more than one image (5% of tweets, 10% of all images). These tweets break down as follows: 2.5% of tweets (5% of all image posts) contained two images, and 2% of tweets and 4% of all images containing three to four images, with four images being the max number of images per tweet on Twitter. The top three keyword categories found within the textual content contained in images for our data set are as follows: 19% of images featured keywords about official response, 18% of all images featured keywords about Surveillance, and 12% of images featured keywords about Efficacy. More information about the lexicon set regarding images can be found in Table 2 in the main article.

*Relationship of Population to Retweet Rates for Local Accounts*

On the suggestion of a reviewer, we investigated whether there was a net difference in retransmission rates for local accounts based on the population of the cities with which the accounts are associated. As our interest is in the possible impact of population *controlling for* other factors, we construct an effective residual retweet rate for each tweet by subtracting the predicted retweet rate from the fitted model to a local estimate of the raw log rate for the tweet in question (specifically, the observed count plus 0.5, corresponding to the posterior mean rate for a locally Poisson model under a Jeffreys prior). This effective residual can be interpreted as the log of the fraction by which each tweet exceeded or failed to achieve the retweet rate that would be expected given its other characteristics (including other account characteristics, as well as posting time). We then relate this residual to the log population within the city of the posting account, allowing us to determine whether there is an association between population size and amplification above and beyond the other factors examined in our model. Overall, we observe a correlation of -0.16 between log population and log retweet residual, a weak but significant (*p*<2.2e-16) relationship indicating that posts by accounts from larger cities are retweeted slightly less frequently (net of all other factors) than posts by accounts from smaller cities.

The detail of this relationship can be better appreciated by plotting the logged residual against log population, as shown in Figure S1. A smoothed mean line (red) has been added to show trend. An examination of the running average reveals that the overall negative correlation is driven entirely by a trend towards negative residuals for cities with sizes above approximately 1.5 million people (see dotted line). Tweets in these locations are retweeted slightly *less* often than one would otherwise expect, although the effect is very small. By contrast, we see no evidence of deviations for smaller cities, down to approximately 43,000 (the lower limit of our data).


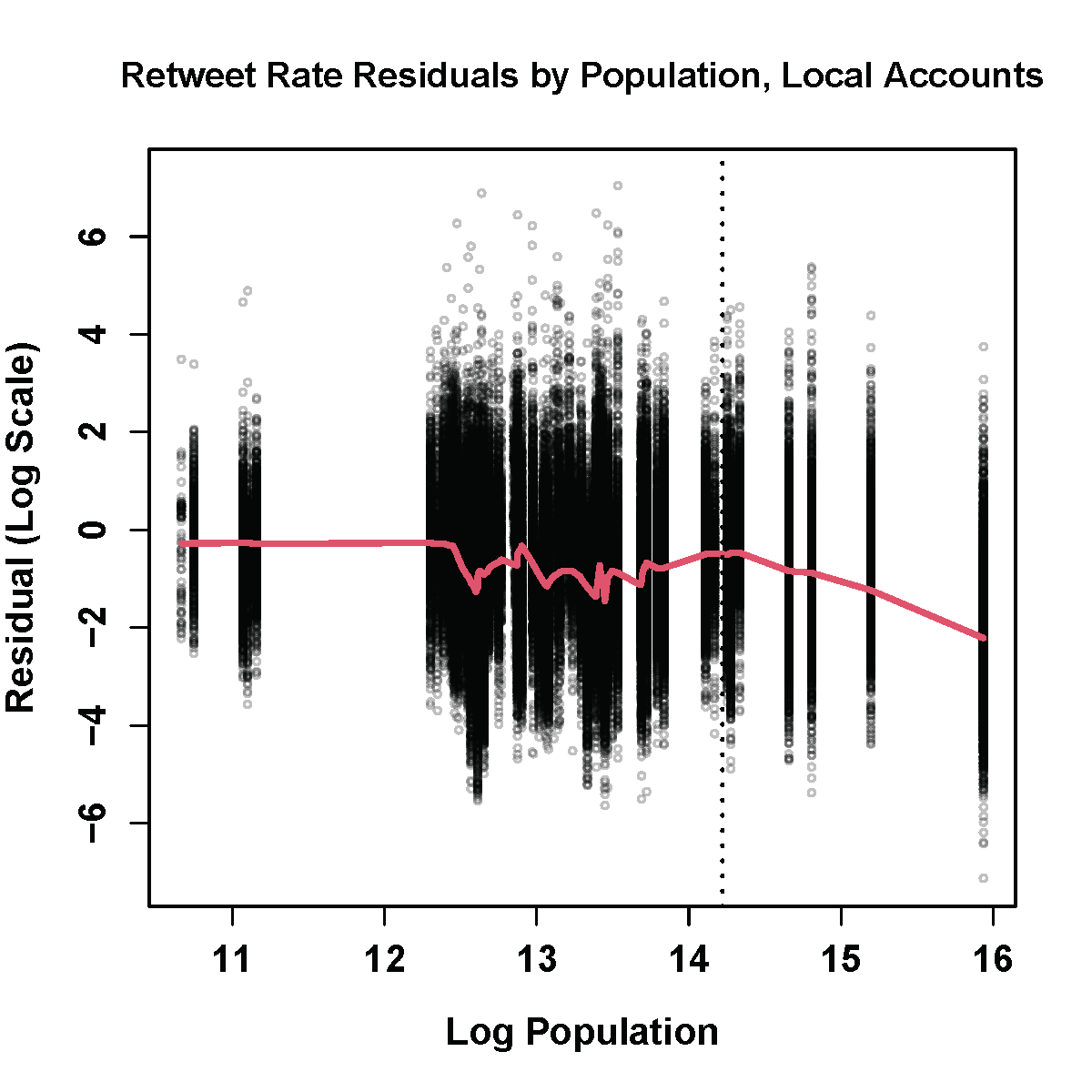


Figure S1. Correlation between Model Residuals and Local Account Populations. Points indicate estimated log residuals per tweet; positive values indicate tweets with higher than expected retweet rates, while negative values indicate tweets with lower than expected retweet rates.  Red line indicates smoothed trend (Friedman’s method).^[[1]](#footnote-1)^  No relationship between city populations and retweet residuals is evident for cities smaller than approximately 1.5 million persons (dotted line).  For populations above 1.5 million, observed retweet rates decline slightly with population size.

While this does not provide definitive evidence against strong differences in retweet rates for accounts from rural areas, it does suggest that any such effect would have to be localized to communities smaller than approximately 40,000 persons (since we see no indication of an effect for small cities). By contrast, it would appear that if there are additional population effects, they instead affect only mega-cities, whose accounts receive somewhat less attention than would otherwise be expected. Further studies examining population effects in greater detail would be required to confirm this apparent relation. However, we do observe that even the mega-city effect is quite small, and unlikely to reshape recommendations for messaging practices.

1. Friedman, J. H. (1984) "A variable span scatterplot smoother." Laboratory for Computational Statistics, Stanford University. Technical Report No. 5. [↑](#footnote-ref-1)
